# The Natural Chemopreventive Agent Sulforaphane Inhibits STAT5 Activity

**DOI:** 10.1371/journal.pone.0099391

**Published:** 2014-06-09

**Authors:** Sophia Pinz, Samy Unser, Anne Rascle

**Affiliations:** Stat5 Signaling Research Group, Institute of Immunology, University of Regensburg, Regensburg, Germany; University of Regensburg, Germany

## Abstract

Signal transducer and activator of transcription STAT5 is an essential mediator of cytokine, growth factor and hormone signaling. While its activity is tightly regulated in normal cells, its constitutive activation directly contributes to oncogenesis and is associated to a number of hematological and solid tumor cancers. We previously showed that deacetylase inhibitors can inhibit STAT5 transcriptional activity. We now investigated whether the dietary chemopreventive agent sulforaphane, known for its activity as deacetylase inhibitor, might also inhibit STAT5 activity and thus could act as a chemopreventive agent in STAT5-associated cancers. We describe here sulforaphane (SFN) as a novel STAT5 inhibitor. We showed that SFN, like the deacetylase inhibitor trichostatin A (TSA), can inhibit expression of STAT5 target genes in the B cell line Ba/F3, as well as in its transformed counterpart Ba/F3-1*6 and in the human leukemic cell line K562 both of which express a constitutively active form of STAT5. Similarly to TSA, SFN does not alter STAT5 initial activation by phosphorylation or binding to the promoter of specific target genes, in favor of a downstream transcriptional inhibitory effect. Chromatin immunoprecipitation assays revealed that, in contrast to TSA however, SFN only partially impaired the recruitment of RNA polymerase II at STAT5 target genes and did not alter histone H3 and H4 acetylation, suggesting an inhibitory mechanism distinct from that of TSA. Altogether, our data revealed that the natural compound sulforaphane can inhibit STAT5 downstream activity, and as such represents an attractive cancer chemoprotective agent targeting the STAT5 signaling pathway.

## Introduction

With an estimated 32.6 million people living with cancer and 8.2 million deaths attributed to cancer worldwide in 2012 [1], cancer prevention has become a public health priority. About a third of all cancer cases are thought to be associated to behavioral and dietary risks and are thus considered preventable [2], [3]. Dietary chemoprevention has gained considerable interest over the past few years as a simple and efficient approach to lower overall cancer risk and reduce cancer incidence and mortality [2], [3]. For dietary chemoprevention strategies to be successful however, a number of conditions have to be met. First, the beneficial nutritional compound must be provided by easily accessible food. Second, its consumption must lead to detectable and reasonable concentrations in the blood while being properly distributed throughout the body to reach target tissues. Finally, since cancer is a multistep process from early carcinogenesis to tumor initiation, promotion and progression, the ability of the dietary molecule to target multiple pathways simultaneously would be advantageous. The isothiocyanate sulforaphane (SFN) found in abundance in cruciferous vegetables such as broccoli fulfill these requirements and is thus viewed as an ideal cancer chemopreventive agent [4]–[6].

Chemopreventive agents are usually classified as blocking and suppressive agents. Blocking agents inhibit phase I enzymes that convert pro-carcinogens into carcinogens and/or induce phase II enzymes that stimulate the detoxification and elimination of carcinogens. Suppressive agents inhibit malignant transformation by targeting pathways controlling cell proliferation, differentiation and survival [7]. Sulforaphane was initially identified as a potent inducer of phase II detoxification enzymes [4], via the Keap1/Nrf2 pathway and as a result of SFN activity as an electrophile reacting with protein thiols [8]–[12]. SFN was thereafter shown to inhibit the activity of phase I enzymes and act as a cancer suppressive agent by modulating signaling pathways involved in cell growth, apoptosis, inflammation and angiogenesis [5], [6]. Interestingly, SFN chemoprotection properties have not only been demonstrated *in vitro* but also *in vivo*, both in animal models and in humans [5], [6]. Several clinical trials investigating the beneficial role of SFN in cancer therapy and prevention are currently ongoing worldwide [13].

Among the cancer suppression functions of SFN, its activity as a histone deacetylase inhibitor is of particular interest for cancer prevention and therapy [14], [15]. Deacetylase inhibitors indeed represent a promising new class of anti-cancer drugs. The deacetylase inhibitor suberoylanilide hydroxamic acid SAHA (Vorinostat) has been approved for the treatment of cutaneous T cell lymphoma and several other deacetylase inhibitors are being currently evaluated in clinical trials for the treatment of various types of cancers [16]. Inhibition of deacetylase activity by SFN was demonstrated in cancer cell lines [17], [18], mice [19], [20], and human subjects [19]. Treatment with SFN results in an increase in acetylated histone H3 and H4 both globally and locally at promoters of genes such as the cell cycle regulator *p21* [17], [18], [20].

We previously showed that the deacetylase inhibitors sodium butyrate, trichostatin A (TSA) and suberoylanilide hydroxamic acid (SAHA) inhibit IL-3-mediated STAT5 transcriptional activity in the mouse pro-B cell line Ba/F3 [21]. STAT5 (signal transducer and activator of transcription 5) is a key regulator of cell proliferation, differentiation and survival [22], [23]. Following stimulation by cytokine, growth factor or hormone, inactive cytosolic STAT5 is phosphorylated by the tyrosine kinase JAK2. Phosphorylated STAT5 then dimerizes, translocates into the nucleus, binds to specific DNA binding sites, and activates transcription of STAT5 target genes (e.g. *Cis, c-Myc*, *Pim-1*, *Osm, Socs-1*) [22], [24]–[26]. STAT5 activity is regulated at multiple levels, through post-translational modifications, protein-protein interactions and tetramerization [22], [27]–[34]. Attenuation of the pathway is tightly regulated via a negative feedback loop mediated by proteins of the SOCS family (CIS, SOCS-1/-3) as well as via dephosphorylation [35], [36]. Improper activation, in particular constitutive activation of STAT5 (caSTAT5) is associated with a broad range of blood and solid tumor cancers [35]–[37]. Constitutive activation of STAT5 directly contributes to oncogenesis through stimulation of cell proliferation and prevention of apoptosis [25], [35]–[38] and is frequently associated to epigenetic silencing of negative regulators of the STAT5 signaling pathway [39]–[43].

STAT5 therefore represents a target of choice for both cancer therapy and prevention [37], [44]–[47]. A number of JAK/STAT inhibitors have been reported. Most of them, whether natural or synthetic small-molecules, target the upstream activating kinase JAK2 [44]–[56], thus inhibiting JAK2-dependent downstream pathways such as MAPK and AKT in addition to STAT5 [57], [58]. Fewer inhibitors have been described that target STAT5 protein itself and its transcriptional activity [21], [59]–[64]. We showed that inhibition of STAT5 activity by deacetylase inhibitors takes place at the transcriptional level. We demonstrated that deacetylase inhibitors target STAT5-mediated transcriptional initiation by preventing recruitment of the basal transcription machinery, without affecting STAT5 activation (phosphorylation) and binding to DNA [21], [65].

Given the central role of STAT5 as a relevant target for cancer chemoprevention and its sensitivity to deacetylase inhibitors, we tested the hypothesis that sulforaphane (SFN) might act as an inhibitor of STAT5 activity. We show here, to the best of our knowledge for the first time, that SFN, similarly to the deacetylase inhibitor TSA, inhibits STAT5 activity in both normal and caSTAT5-transformed cells. Like TSA, SFN treatment inhibited STAT5-mediated induction of target genes at the RNA level, without affecting STAT5 initial activation (phosphorylation) and DNA recognition. By contrast to TSA however, this inhibitory effect was not associated with changes in global histone acetylation levels, nor did it affect histone acetylation at specific target genes, thus suggesting a deacetylase-independent effect. Our data uncover STAT5 as a novel molecular target of SFN, hence confirming this dietary isothiocyanate as a potent anti-cancer agent.

## Materials and Methods

### Chemicals

Dimethyl sulfoxide (DMSO) and trichostatin A (TSA) were purchased from SIGMA (D2650 and T8552 respectively). R,S-Sulforaphane (SFN) was from LKT Laboratories (S8044) and Imatinib was from Cayman Chemical (No. 13139). Compounds were dissolved in DMSO at a final concentration of 1 mM (TSA), 5 mM (Imatinib) or 100 mM (SFN).

### Cell lines and drug treatments

All cell lines were grown in RPMI 1640 (PAN-Biotech P04-16500) supplemented with 10% heat-inactivated fetal calf serum (FCS; PAN-Biotech), penicillin/streptomycin (PAN-Biotech) (thereafter designated as RPMI-based medium) and cultivated at 37°C under 5% CO_2_ in a humidified incubator. K562 cells (a kind gift from Daniela Männel, University of Regensburg, Germany; [66]) were maintained in RPMI-based medium. The non-tumorigenic immortalized interleukin-3 (IL-3)-dependent mouse pro-B cell line Ba/F3 (a kind gift from Jacqueline Marvel, IFR 128 BioSciences Gerland-Lyon Sud, France; [67]) was grown in RPMI-based medium supplemented with 2 ng/mL rmIL-3 (ImmunoTools). The Ba/F3-1*6 cell line (clone F7) stably expressing the constitutively active mouse STAT5A-1*6 mutant [68] was generated as previously described [56] and grown in RPMI-based medium supplemented with 500 µg/mL G418.

For cytokine stimulation of Ba/F3 cells, cells were washed twice in RPMI 1640 and rested in RPMI-based medium for 9 to 12 hours before addition of 5 ng/mL IL-3 for 30-60 minutes, as indicated in the figure legends. Inhibitor (TSA, SFN) or vehicle (DMSO) was added 30 minutes prior to IL-3 stimulation. Ba/F3-1*6 and K562 cells were treated for 60-90 minutes with TSA, SFN or DMSO (vehicle), as indicated. With the exception of the cell viability assays, DMSO final concentration was adjusted to 0.1% (cytotoxicity assay) or to 0.02% (other assays) in all conditions.

### Gene expression analysis by quantitative RT-PCR

Quantitative RT-PCR was performed as previously described [56]. Data were normalized to mouse S9 ribosomal (Ba/F3 and Ba/F3-1*6 cell lines) or human Lamin A/C (LMNA) (K562 cell line) mRNAs, and expressed as relative mRNA levels, as previously described [21], [24], [56], [65]. Mouse- and human-specific real-time PCR primers used in this study have been already reported [21], [24], [56], [69]. Data are mean ±SD of the quantitative PCR, performed in either duplicate or triplicate, and are representative of at least three independent experiments. Raw data (CT values) are available in File S1.

### Cytotoxicity assays

WST-1 assays (11 644 807 001, Roche) were performed as recently reported [56] to monitor changes in metabolically active mitochondrial dehydrogenases as a result of TSA- or SFN-induced cytotoxicity. Briefly, rested Ba/F3 and growing Ba/F3-1*6 and K562 cells were pre-treated for 30 minutes with the indicated concentrations of TSA, SFN or DMSO (vehicle), WST-1 reagent was added to the cells either alone (Ba/F3-1*6, K562) or together with IL-3 (Ba/F3), and absorbance was measured after 90 minutes (maximal duration of inhibitor treatment in our gene expression assays) in a microplate reader (Mithras LB 940, Berthold Technologies; 450/620 nm). A positive control for no mitochondrial enzyme activity (1% Triton X-100) was included in every experiment. Data are expressed as a percentage of cytotoxicity relative to DMSO (vehicle). Data shown are representative of two independent experiments. Raw data (OD 450/620 nm) are available in File S1.

### Cell viability assays

The number of living and dead cells was evaluated by Trypan Blue exclusion after 24 and 48 hours of TSA or SFN treatment, as previously described [56]. Viable cell number for each treatment, reflecting cell proliferation and survival, is expressed as a function of time. Data shown are representative of at least two to three independent experiments.

### Quantitative chromatin immunoprecipitation (ChIP) assays

Chromatin immunoprecipitation was performed as previously described [56]. Antibodies specific for STAT5, RNA polymerase II, acetylated histone H3 (Ac-H3) and acetylated histone H4 (Ac-H4) have been reported [21]. ChIP grade anti-histone H3 antibody (total H3) was from abcam (ab1791). Real-time PCR primers specific for the STAT5 binding sites and for the transcription start site (tss) of the mouse *Cis* gene (amplicons A and B) have been described [21]. Forward and reverse real-time PCR primers specific for amplicons C to H along the open reading frame of the mouse *Cis* gene were respectively: amplicon C, 5′-GGACTTCGAGTGGTGTGCCTA-3′ and 5′-GGCTCCGTTTCCCTATCCA-3′; amplicon D, 5′-CATTCCTCCGTCCCAGGTC-3′ and 5′-ACCTCAGGCTGGCTTCCTAAG-3′; amplicon E, 5′-AATTTTCGGACTCTTCGGCA-3′ and 5′-CACCCAAGAAAGGAAGGCAG-3′; amplicon F, 5′-CAGCTCCTAACCACCCCTGTT-3′ and 5′-ACTGGCTGGGAAAGGCAAC-3′; amplicon G, 5′-GAGGACACTGCCTTCCCTCA-3′ and 5′-AAGCTTCTACCCACTCCGGC-3′; amplicon H, 5′-TACCCCTTCCAACTCTGACTGAGC-3′ and 5′-TTCCCTCCAGGATGTGACTGTG-3′. Real-time PCR primers specific for the STAT5 binding sites and for the tss of the mouse *Osm* gene (amplicons I and J) have been described [21], [56]. Forward and reverse real-time PCR primers specific for the mouse *p21* proximal promoter region (−120/−61 relative to the tss; amplicon K) were 5′-GAGGGCGGGCCAGCGAGTC-3′ and 5′-CTCAGAGGCAGGACCAACCCACTC-3′. Data are mean ±SD of the quantitative PCR, performed in either duplicate or triplicate, and are representative of at least two to three independent experiments. Raw data (CT values) are available in File S1.

### Protein analysis by Western blot

Whole-cell Brij protein lysis (analysis of STAT5 activation) and immunoblotting were performed as described [21], [56], [69]. Antibodies used for the detection of pSTAT5, STAT5A, STAT5B, STAT5A+B, α-tubulin, Anti-Rabbit and Anti-Mouse IgG-Peroxidase, as well as their respective working dilutions, have been reported [56].

Whole-cell protein lysis for the analysis of histone acetylation was performed as follows. Equal number of growing Ba/F3 cells cultured in the presence of 0.2 µM TSA or 10 µM SFN were harvested at the indicated times and resuspended in Freeze-Thaw lysis buffer (600 mM NaCl, 20 mM Tris-HCl pH 8.0, 20% Glycerol, protease inhibitors). Upon three freeze-thaw cycles, whole-cell lysates were treated with DNase I (0.1 µg/µl final in the presence of 5 mM MgCl_2_) for 45 minutes at +4°C, adjusted to 1× Laemmli buffer containing β-mercaptoethanol and heated at 95°C for 10 minutes. Denatured samples were centrifuged 15 minutes at maximum speed to eliminate cell debris, before loading equal volumes (corresponding to equal cell number) on a 15% SDS-PAGE for Western blot analysis. Antibodies used for the detection of histone proteins were: Anti-acetylated histone H3 (06-599, Upstate/Millipore; 1:5000), Anti-acetylated histone H4 (06–866, Upstate/Millipore; 1:2000) and Anti-histone H3 (ab1791, Abcam; 1:3000).

Apparent molecular weight of detected proteins was as predicted by the antibody manufacturers, i.e. 90 kDa for STAT5 (with STAT5A running slightly slower than STAT5B in SDS-PAGE), 55 kDa for α-tubulin, 17 kDa for histone H3 and 11 kDa for histone H4. Immunoblots shown are representative of at least three independent experiments.

## Results

### Sulforaphane treatment inhibits IL-3-mediated expression of STAT5 target genes in Ba/F3 cells

**Figure 1:**
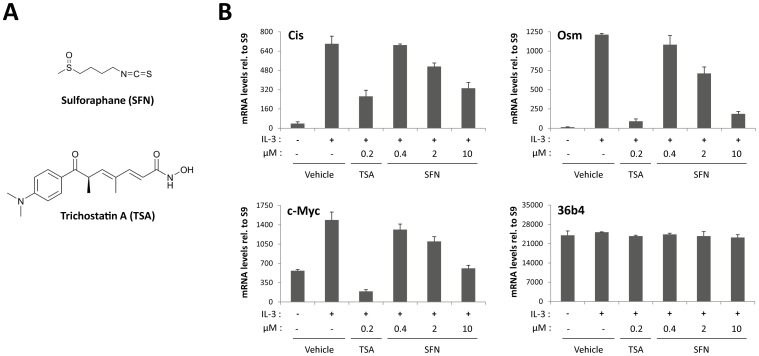
Sulforaphane (SFN) treatment inhibits IL-3-mediated induction of STAT5 target genes in Ba/F3 cells in a dose-dependent manner. (**A**) Structure of the natural compound sulforaphane (SFN) and of the synthetic deacetylase inhibitor trichostatin A (TSA) used in this study. (**B**) Ba/F3 cells were pre-treated 30 minutes with DMSO (vehicle), 0.2 µM TSA, 0.4, 2 or 10 µM SFN and further stimulated 30 minutes with 5 ng/mL IL-3. Following cell harvest, expression of the STAT5 target genes *Cis, Osm*, *c-Myc* and of the housekeeping gene *36b4* were measured by quantitative RT-PCR, as described in Materials and Methods. Similarly to TSA, SFN inhibits IL-3-mediated induction of STAT5-regulated genes.

The effect of sulforaphane (SFN; Figure 1A) on the STAT5 signaling pathway was investigated in the IL-3-dependent Ba/F3 cell line. Expression of the STAT5 target genes *Cis*, *Osm* and *c-Myc* upon IL-3 stimulation was monitored by quantitative RT-PCR in cells pre-treated with increasing amount of SFN (0.4-10 µM) or with 0.2 µM trichostatin A (TSA). TSA was used as a reference inhibitor throughout this study (Figure 1A), in accordance with our previous observation that TSA inhibits STAT5-mediated transcription [21], [65]. Similarly to TSA, SFN treatment was able to inhibit IL-3-mediated induction of the STAT5 target genes *Cis*, *Osm* and *c-Myc* in a dose-dependent manner, while expression of the housekeeping gene *36b4* remained unaffected (Figure 1B).

**Figure 2:**
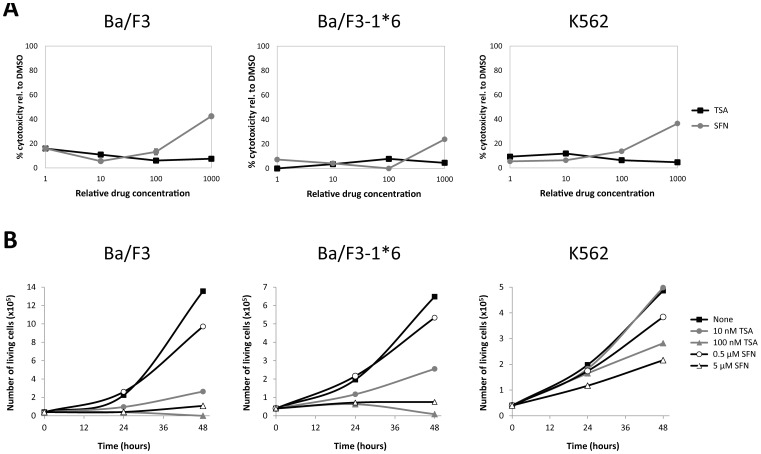
Effect of SFN treatment on cytotoxicity and viability of normal (Ba/F3) and transformed (Ba/F3-1*6, K562) cells. (**A**) The WST-1 reagent was added to cells following 30 minutes of pre-treatment with 0.001, 0.01, 0.1 and 1 µM TSA or with 0.1, 1, 10 and 100 µM SFN. IL-3 (5 ng/mL) was supplemented to rested Ba/F3 cells at the same time as the WST-1 reagent to mimic the IL-3 stimulation conditions used in other assays. OD measurement was performed after 90 minutes incubation with the WST-1 reagent, and the percentage of cytotoxicity was normalized to the vehicle control. (**B**) Growing Ba/F3, Ba/F3-1*6 and K562 cells were incubated for 24 and 48 hours in the presence of the indicated concentrations of TSA and SFN. Cell viability was measured by Trypan Blue exclusion assay.

We next verified that the inhibitory effect of SFN was not the result of cytotoxicity. WST-1 assays were performed in IL-3-stimulated Ba/F3 cells using concentrations of 0.1–100 µM SFN (Figure 2A). No cytotoxicity was detected up to 10 µM SFN while 40% toxicity was noted in the presence of 100 µM (Figure 2A). SFN was used thereafter at concentrations not exceeding 10–20 µM. The effect of SFN on cell proliferation and survival of IL-3-growing Ba/F3 cells was also monitored (Figures 2B and S1). Like TSA, SFN affected cell growth and viability in a dose-dependent manner. Ba/F3 cells cultured for 24 and 48 hours in the presence of 5 µM SFN stopped dividing (Figure 2B) and partially died (30% dead cells monitored upon trypan blue staining; Figure S1) while cells grown in 0.5 µM SFN showed limited inhibition of cell proliferation and no cell death (Figures 2B and S1 respectively). Ba/F3 cells grown in the presence of 100 nM TSA stopped dividing and died, whereas strong cell growth inhibition but limited cell death was monitored at 10 nM TSA (Figures 2B and S1).

### Sulforaphane treatment inhibits constitutive STAT5 activity in transformed cell lines

To further characterize the effect of SFN on STAT5 signaling, expression of a series of STAT5 target genes (*Cis*, *c-Myc, Pim-1, Socs-1, Osm*) and STAT5-independent genes (*JunB*, *c-Fos*, *36b4*) was analyzed in cells showing regulated STAT5 activity (IL-3-stimulated Ba/F3 cells) and cells transformed upon expression of constitutive active STAT5 (Ba/F3-1*6, K562). Ba/F3-1*6 cells stably express a mutant form of mouse STAT5A (so-called 1*6) carrying two amino acids substitutions which result in constitutive STAT5 phosphorylation, nuclear localization and transactivation properties [68]. Expression of STAT5A-1*6 confers IL-3-independent growth to Ba/F3 cells *in vitro* and tumorigenicity to bone marrow cells *in vivo* [68], [70]. In the absence of IL-3, the upstream activating kinase JAK2 is not activated [25], [68]. Therefore, pathways downstream of JAK2, such as MAPK and AKT [57], [58], are not activated in Ba/F3-1*6 cells, in contrast to IL-3-stimulated Ba/F3 cells. The mechanism of constitutive activation of STAT5A-1*6 is unclear. Constitutive phosphorylation by basal JAK2 activity or by an unidentified tyrosine kinase, and increased stability of phospho-STAT5A-1*6 have been proposed to contribute to its constitutive activity [25], [68]. The human K562 leukemia cell line expresses a constitutively active BCR-ABL tyrosine kinase. BCR-ABL oncogenic fusion constitutively phosphorylates STAT5 proteins, directly contributing to oncogenesis [71]–[73].

First, the effect of SFN on cytotoxicity and cell viability of Ba/F3-1*6 and K562 cells was evaluated, as done before in Ba/F3 cells (Figures 2 and S1). SFN-mediated cytotoxicity in Ba/F3-1*6 and K562 cells was comparable to that observed in Ba/F3 cells and only detectable at a concentration of 100 µM (Figure 2A). The effect of SFN and TSA on cell proliferation and survival was comparable in the STAT5-1*6-transformed Ba/F3 cells and in the untransformed parental cell line Ba/F3. The human leukemic cell line K562 exhibited a reduced sensitivity to both SFN and TSA as revealed by the limited effect on cell proliferation and cell death (Figures 2B and S1).

**Figure 3:**
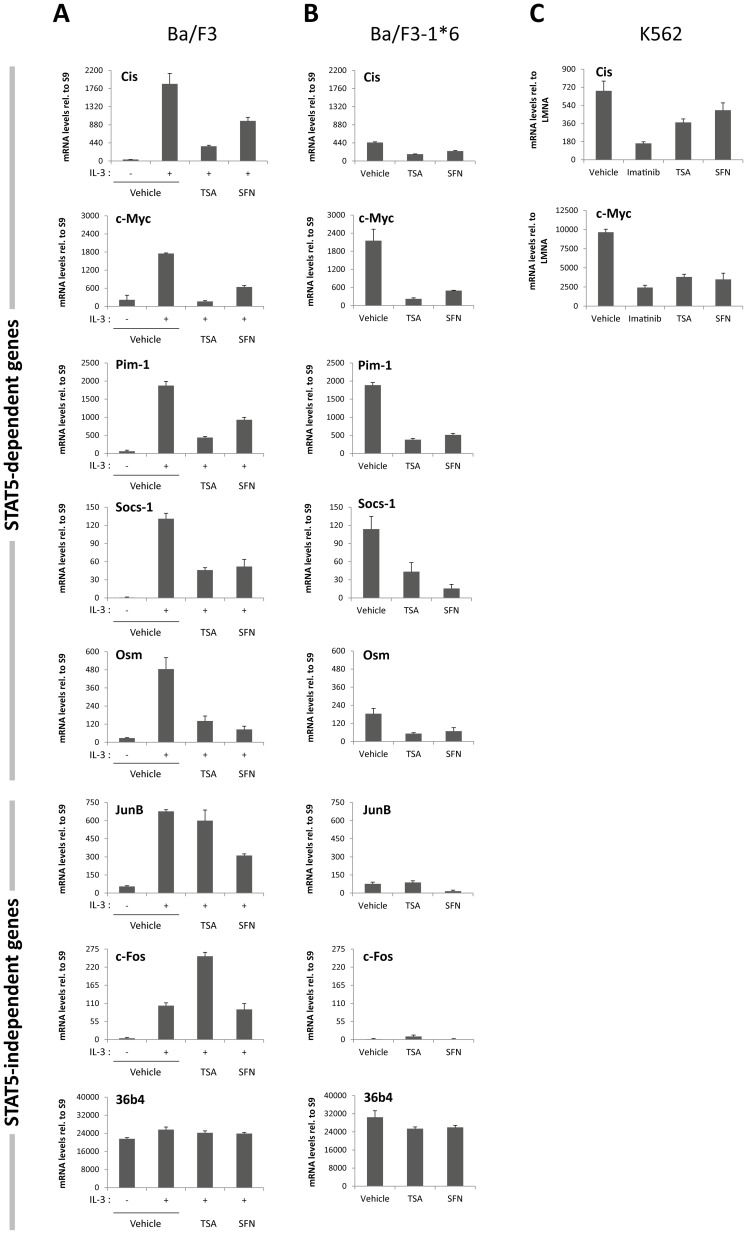
SFN treatment inhibits STAT5 constitutive activity in the transformed cell lines Ba/F3-1*6 and K562. Ba/F3 (**A**), its transformed counterpart Ba/F3-1*6 (**B**) and human leukemic K562 (**C**) cells were treated 90 minutes with DMSO (vehicle), 0.2 µM TSA, 10 µM SFN or 1 µM Imatinib. Ba/F3 cells (**A**) were stimulated with 5 ng/mL IL-3 for 60 minutes following 30 minutes of drug pre-treatment. Expression of STAT5-dependent (*Cis*, *c-Myc, Pim-1, Socs-1, Osm*,) and -independent (*JunB*, *c-Fos*, *36b4*) genes was analyzed by quantitative RT-PCR. Gene expression data were normalized to cDNA levels derived from mouse ribosomal *S9* (**A, B**) or human Lamin A/C (*LMNA*) (**C**) mRNAs. (**A, B**) The Y-axis scales were adjusted to allow a direct comparison of relative expression levels in Ba/F3 and Ba/F3-1*6 cells.

Ba/F3, Ba/F3-1*6 and K562 cells were treated with vehicle, 0.2 µM TSA or 10 µM SFN for 90 minutes. After 30 minutes of inhibitor pre-treatment, Ba/F3 cells were additionally stimulated 60 minutes with IL-3. K562 cells were also treated with 1 µM Imatinib, a specific inhibitor of the BCR-ABL tyrosine kinase, as a positive control for inhibition of constitutive STAT5 phosphorylation in K562 cells [74]. Expression of STAT5 target genes (*Cis*, *c-Myc, Pim-1, Socs-1, Osm*), of JAK2/MAPK-regulated STAT5-independent genes (*JunB*, *c-Fos*) [75], [76], and of a housekeeping control gene (*36b4*) was evaluated by quantitative RT-PCR (Figure 3).

As predicted upon expression of constitutively active STAT5-1*6 [21], [25], expression of all STAT5 target genes investigated was up-regulated to various extents in growing Ba/F3-1*6 cells in comparison to unstimulated Ba/F3 cells (Figure 3A–B). As anticipated, expression of all STAT5 target genes was inhibited by TSA in all three cell lines (Figure 3A–C), as was expression of *Cis* and *c-Myc* in Imatinib-treated K562 cells (Figure 3C). Likewise, expression of all STAT5-target genes investigated was inhibited by SFN in Ba/F3, Ba/F3-1*6 and K562 cells, demonstrating that SFN is able to inhibit both regulated and constitutive STAT5 activity.

The specificity of action of SFN was further assessed by monitoring expression of the MAPK-regulated genes *JunB* and *c-Fos*. While expression of *JunB* and *c-Fos* was induced by IL-3 in Ba/F3 cells, their expression remained at background levels in Ba/F3-1*6 cells (Figure 3A-B), as expected from the absence of JAK2/MAPK activation in Ba/F3-1*6 cells [25]. Interestingly, expression of *JunB* and *c-Fos* was differentially affected by SFN in IL-3-stimulated Ba/F3 cells. While *c-Fos* expression remained unaffected, expression of *JunB* was reduced upon SFN treatment (Figure 3A). This contrasts with the effect of TSA which did not affect *JunB* expression while up-regulating *c-Fos* expression, in agreement with our previous data [21]. The observation that expression of *JunB* but not *c-Fos* is affected by SFN suggests that it does not target the JAK2/MAPK pathways itself. This is comforted by the observation that *JunB* basal expression in Ba/F3-1*6 cells was also reduced upon SFN treatment (Figure 3B). The observation that SFN and TSA exert both redundant and non-redundant effects on gene expression also suggest that they exhibit overlapping but also distinct activities.

### Sulforaphane treatment does not alter STAT5 phosphorylation

**Figure 4:**
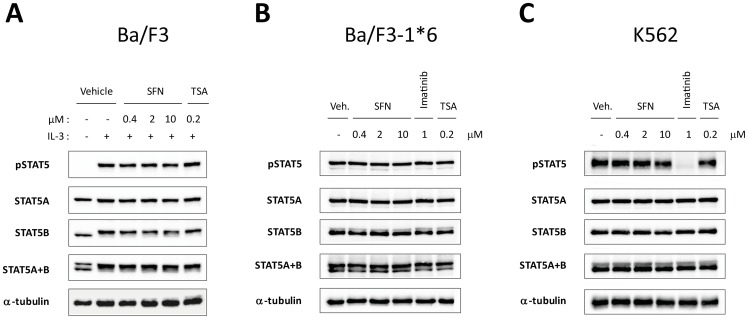
SFN treatment does not affect STAT5 phosphorylation. Ba/F3 (**A**), Ba/F3-1*6 (**B**) and K562 (**C**) cells were treated 60 minutes with DMSO (vehicle) or the indicated concentrations of TSA, SFN or Imatinib. Ba/F3 cells (**A**) were stimulated with 5 ng/mL IL-3 for 30 minutes following 30 minutes of drug pre-treatment. Whole-cell Brij protein lysates were analyzed by Western blot using antibodies specific for phospho-STAT5 (pSTAT5), STAT5A, STAT5B, STAT5A and B, and α-tubulin (loading control).

To C pathway is inhibited by SFN, the phosphorylation status of STAT5 was evaluated in Ba/F3, Ba/F3-1*6 and K562 cells treated with SFN. STAT5 proteins are encoded by two highly related genes, STAT5A and STAT5B, with both redundant and unique functions [22], [24]. STAT5 phosphorylation and STAT5A and STAT5B protein levels were investigated by Western blot using a phospho-STAT5-specific antibody (pSTAT5), and STAT5A- and STAT5B-specific antibodies respectively (Figure 4). Ba/F3, Ba/F3-1*6 and K562 cells were treated with 0.2 µM TSA or 0.4–10 µM SFN for 60 minutes. After 30 minutes of inhibitor pre-treatment, Ba/F3 cells were stimulated 30 minutes with IL-3. Ba/F3-1*6 and K562 cells were also treated with 1 µM Imatinib, as a positive control for pSTAT5 inhibition in K562 cells. The BCR-ABL inhibitor Imatinib drastically and specifically inhibited STAT5 phosphorylation in K562 cells (Figure 4C), as previously reported [56], [74]. In agreement with our previous data in Ba/F3 cells [21], TSA did not affect STAT5 phosphorylation in any of the three cell lines (Figure 4A–C). Likewise, SFN treatment had no effect on STAT5 phosphorylation or on STAT5A/B protein levels (Figure 4A–C). These results indicate that, similarly to TSA, SFN does not inhibit the initial activation of STAT5 and rather suggest a downstream inhibitory event. Consequently, we analyzed the effect of SFN treatment on STAT5-mediated transcription.

### Sulforaphane treatment inhibits STAT5-mediated transcriptional activity

**Figure 5:**
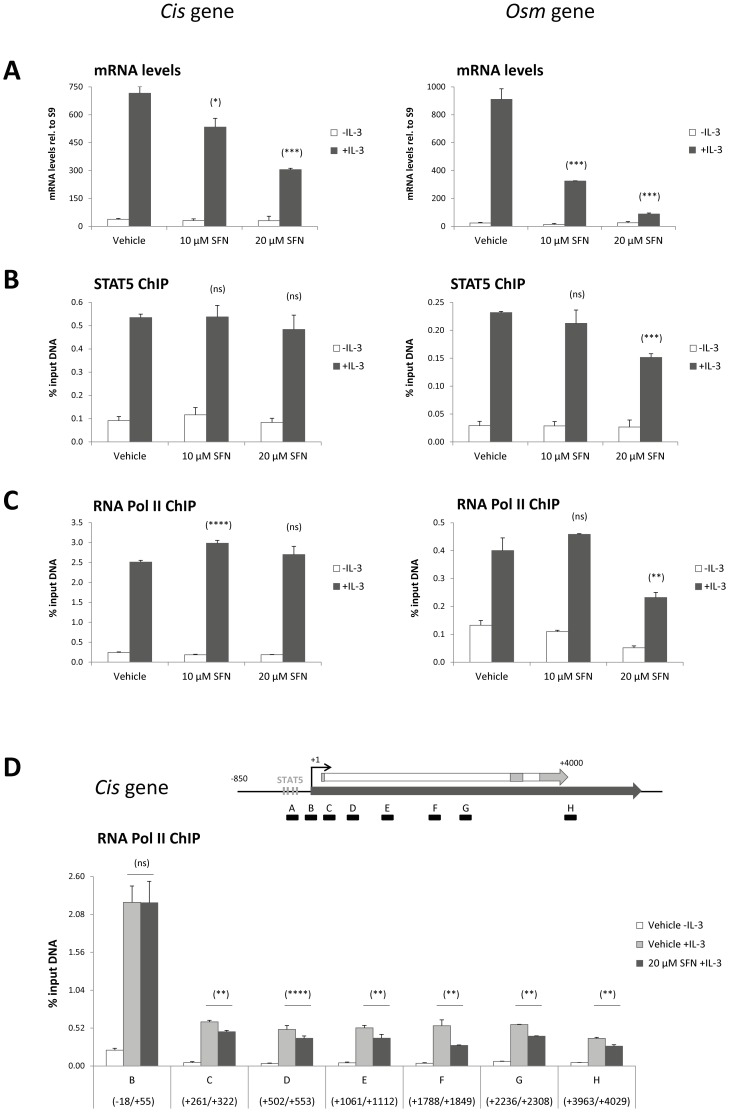
STAT5 binding and RNA polymerase II recruitment to the promoter of STAT5 target genes are marginally affected by SFN treatment. Ba/F3 cells were pre-treated 30 minutes with DMSO (vehicle), 10 µM or 20 µM SFN and further stimulated with 5 ng/mL IL-3 for 30 minutes. Cells were harvested for both gene expression analysis of the *Cis* and *Osm* genes by quantitative RT-PCR (A) and for chromatin immunoprecipitation (ChIP) (B–D). ChIP was performed using antibodies directed against STAT5 (B) or RNA polymerase II (RNA Pol II; C, D) proteins. Co-precipitated genomic DNA was analyzed by quantitative PCR using primers specific for the STAT5 binding sites (amplicons A and I in Figure S2) (STAT5 ChIP; B) or the transcription start site (amplicons B and J in Figure S2) (RNA Pol II ChIP; C) of the mouse *Cis* and *Osm* genes, as well as with primers spanning the open reading frame of the *Cis* gene (RNA Pol II ChIP; D). Schematic representation of the *Cis* gene with its transcribed region (dark grey arrow), the coding sequence (white arrow with exons in light grey), the four STAT5 binding sites within its proximal promoter region, and the quantitative PCR amplicons investigated (A to H-labeled black boxes) is shown in (D). The RNA polymerase II occupancy along the transcribed region of the *Cis* gene is slightly but consistently reduced in SFN-treated cells. Two-tailed paired Student's t-test, SFN-treated compared to vehicle control (IL-3-stimulated); *P<0.05, **P<0.005, ***P<0.001, ****P<0.0001; ns, not significant.

We previously showed that the deacetylase inhibitor TSA inhibits STAT5-mediated transcription at a step subsequent to STAT5 binding to its target genes by preventing recruitment of the transcriptional machinery [21]. Chromatin immunoprecipitation assays were performed from TSA- and SFN-treated Ba/F3 cells, using antibodies directed against STAT5 and RNA polymerase II proteins. Co-precipitated DNA was examined by quantitative PCR using primers specific for the STAT5 binding sites and transcription start sites respectively of the STAT5 target genes *Cis* and *Osm* (Figure S2). The mouse *Cis* and *Osm* genes are well characterized STAT5 target genes, bearing four and two STAT5 binding sites respectively within their proximal promoter [21], [24], [65], [77], [78] (Figure S2). Pre-treatment of Ba/F3 cells with 10 µM and 20 µM SFN, while leading to reduced Cis mRNA levels, did not affect STAT5 binding or RNA polymerase II recruitment to the *Cis* gene (Figures 5A–C and S3A). The *Osm* gene which is more strongly inhibited by SFN at the mRNA level displayed a partial but dose-dependent decrease in STAT5 and RNA polymerase II recruitment upon SFN treatment (Figures 5A–C and S3B). The marginal effect of SFN on STAT5 association with DNA is similar to that of TSA on the same target genes [21] (Figure S3). However, the absence or modest effect of SFN on RNA polymerase II recruitment at *Cis* and *Osm* genes respectively is in sharp contrast to the previously described effect of TSA, which abolished RNA polymerase II recruitment at both target genes [21] (Figure S3).

Impaired recruitment of RNA polymerase II at the transcription start site of the *Osm* gene correlates well with - und thus might account for - the observed reduction in Osm mRNA level. In contrast, the unchanged occupancy of RNA polymerase II at the transcription start site of the *Cis* gene suggests a further downstream inhibitory event. Transcription is controlled at multiple levels. The RNA polymerase II (Pol II) recruitment step is followed by another critical regulatory event known as promoter-proximal Pol II pausing, and subsequent promoter escape into productive elongation [79]–[82]. Promoter-proximal Pol II pausing is typically found at transcriptionally active and rapidly induced genes and is characterized by a higher RNA polymerase II density at the 5′ end of the gene [79]–[81]. To investigate whether SFN might interfere with transcription elongation, RNA polymerase II occupancy along the open reading frame of the *Cis* gene was monitored by chromatin immunoprecipitation and quantitative PCR analysis of amplicons spread along the mouse *Cis* gene locus (Figure 5D). Upon IL-3 stimulation, RNA polymerase II was uniformly distributed along the *Cis* open reading frame (+261 to +4029) with an increased occupancy around the transcription start site (−18/+55) (Figure 5D), indicative of promoter-proximal Pol II pausing. In Ba/F3 cells pre-treated with SFN, RNA polymerase II remained evenly distributed along the *Cis* transcribed region, arguing against abortive transcription elongation. However, the level of RNA polymerase II, although unchanged at the transcription start site, was slightly but consistently and reproducibly reduced throughout the transcribed region (22% to 49% reduction depending on the amplicon analyzed) (Figures 5D and S4). This overall reduced Pol II association with the *Cis* transcribed region might be indicative of impaired promoter clearance. Whether the reduced Pol II occupancy is sufficient to account for the reduced Cis mRNA level observed in SFN-treated cells (47% reduction in mRNA level; Figure 5A) remains however to be demonstrated.

Altogether, our chromatin immunoprecipitation experiments reveal that both TSA and SFN inhibit transcription of STAT5 target genes at a step following the association of STAT5 with DNA. However, while TSA is able to abrogate RNA polymerase II recruitment to STAT5 target promoters, SFN only partially impaired RNA polymerase II recruitment and/or promoter clearance, therefore suggesting that both small-molecules inhibit transcription through distinct mechanisms.

### Histone acetylation is not affected in SFN-treated Ba/F3 cells

**Figure 6:**
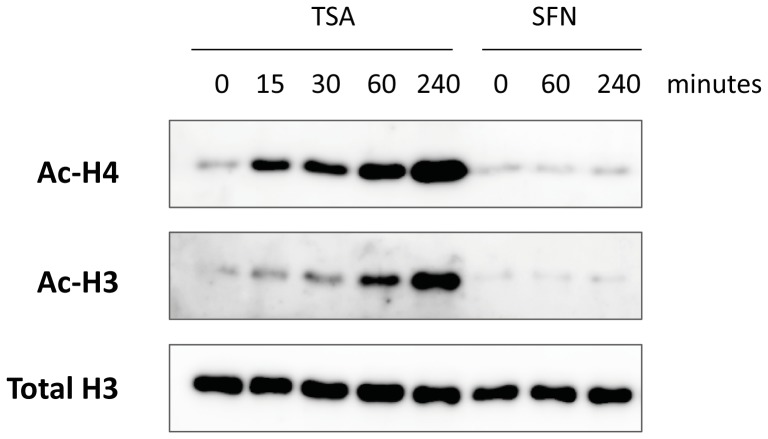
SFN treatment does not affect global histone acetylation level in Ba/F3 cells. Ba/F3 cells were treated for the indicated times with either 0.2 µM TSA or 10 µM SFN. Whole-cell Freeze-Thaw protein lysates were analyzed by Western blot using antibodies specific for acetylated histone H3 (Ac-H3) and H4 (Ac-H4) and for total histone H3 proteins as a reference. While global histone acetylation was markedly increased in cells treated with TSA, no apparent effect was detected upon SFN treatment.

SFN was shown to inhibit histone deacetylase activity in various cell lines [14], [15], [17], [18]. In order to determine whether the inhibitory effect of SFN on STAT5 activity involves inhibition of histone deacetylase activity, the effect of SFN on global histone H3 and H4 acetylation in Ba/F3 cells was investigated. IL-3-growing Ba/F3 cells were treated with 0.2 µM TSA or 10 µM SFN for increasing periods of time up to 4 hours. Histone proteins from whole-cell extracts were analyzed by Western blot using antibodies specific for acetylated histone H3 and H4 and for total histone H3 (Figure 6). Treatment of Ba/F3 cells with TSA for as short as 15 minutes led to a detectable increase in histone H3 and H4 acetylation, which kept increasing over time. To our surprise, treatment with 10 µM SFN had no effect on global histone H3 and H4 acetylation levels, even after 4 hours of treatment, which is beyond the duration of SFN treatment in our gene expression assays (Figure 6). Prolonged SFN treatment up to 48 hours revealed a slight increase in histone H3 acetylation (3.4-fold of untreated control; Figure S5).

**Figure 7:**
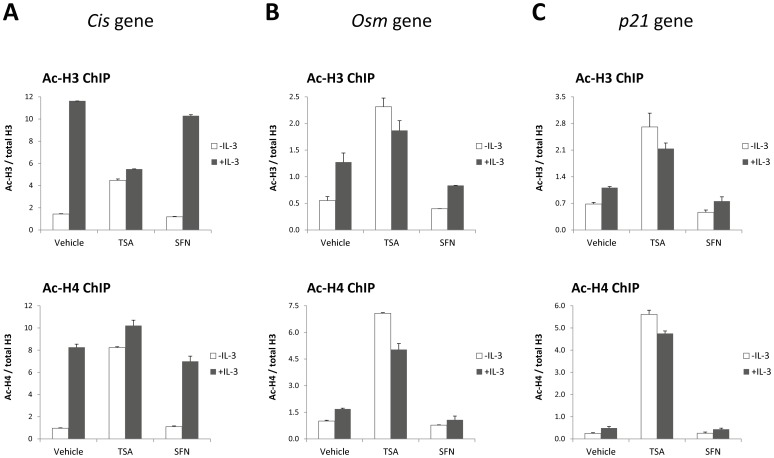
SFN treatment does not affect histone acetylation at the promoters of STAT5 target (*Cis, Osm*) and control (*p21*) genes. Ba/F3 cells were pre-treated 30 minutes with DMSO (vehicle), 0.2 µM TSA or 10 µM SFN and further stimulated 30 minutes with 5 ng/mL IL-3. Chromatin immunoprecipitation (ChIP) was performed using antibodies directed against acetylated histone H3 (Ac-H3) and H4 (Ac-H4) and against histone H3 proteins (total H3). Co-precipitated genomic DNA was analyzed by quantitative PCR using primers specific for the transcription start sites of the mouse *Cis* (**A**) and *Osm* (**B**) genes (amplicons B and J respectively in Figure S2), as well as for the proximal promoter region of the mouse *p21* gene (amplicon K in Figure S2) as a control (**C**). Ac-H3 and Ac-H4 ChIP data were normalized to total Histone H3, to more accurately estimate histone acetylation levels at the investigated gene loci. Corresponding raw ChIP data for Ac-H3, Ac-H4 and H3 immunoprecipitations (expressed as % of input DNA) are shown in Figure S6. While histone acetylation levels were dramatically affected by TSA at all three gene loci, no major change in histone H3 and H4 acetylation was monitored in SFN-treated cells.

Since SFN was shown to alter histone acetylation locally, in particular at the *p21* promoter [17], [18], the level of histone H3 and H4 acetylation at the promoters of the *p21* gene as well as of the STAT5 target genes *Cis* and *Osm* was monitored by chromatin immunoprecipitation (Figure S2). Ba/F3 cells were pre-treated with 0.2 µM TSA or 10 µM SFN and stimulated with IL-3 as before and subjected to chromatin immunoprecipitation using antibodies specific for acetylated histone H3 and H4 and for total histone H3. To better reflect changes in histone acetylation, and because histone H3 occupancy itself changes upon drug treatment and IL-3 stimulation (Figure S6), histone H3 and H4 acetylation data were normalized to total histone H3 levels (Figure 7). Major changes in histone H3 and H4 acetylation levels were observed at *Cis*, *Osm* and *p21* proximal promoter regions upon TSA treatment (Figure 7), in agreement with the global effect of TSA on histone acetylation (Figure 6). In fact histone H3 occupancy itself was greatly affected by TSA, especially at the promoters of unstimulated genes (Figure S6). By contrast, no apparent modifications in histone acetylation (Figure 7) or histone association (Figure S6) at the investigated gene loci were noticeable in SFN-treated cells.

In the whole, while treatment of Ba/F3 cells with TSA resulted in changes in histone acetylation and association at the investigated genes, no such changes were detectable upon SFN treatment. Together with the observation that RNA polymerase II association and/or stability was also differentially affected by both inhibitory agents, our data support the idea that TSA and SFN inhibit transcription of STAT5 target genes through distinct mechanisms.

## Discussion

**Figure 8:**
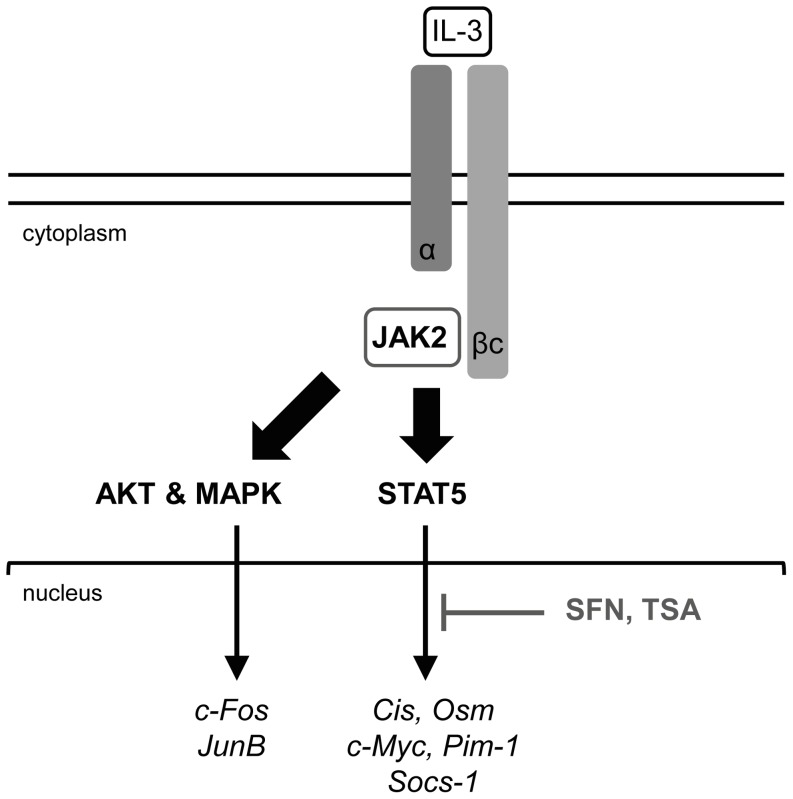
Model of inhibition of STAT5 activity by SFN in Ba/F3 cells. IL-3 binding to its receptor leads to activation of the receptor-associated JAK2 tyrosine kinase. In turn, JAK2 activates the downstream STAT5, MAPK and AKT pathways via phosphorylation (broad arrows), resulting in induced transcription of downstream target genes (thin arrows). We showed that, similarly to TSA, SFN inhibits induction of STAT5 target genes without interfering with STAT5 initial activation (phosphorylation) and binding to DNA. In contrast to TSA however, SFN does not affect histone acetylation, neither globally nor locally at specific gene loci, and only moderately interferes with recruitment of the transcriptional machinery, suggesting an alternative mechanism of transcriptional inhibition, independent of deacetylase activity. JunB expression was also inhibited by SFN in Ba/F3 cells, although via a MAPK-independent mechanism.

The natural isothiocyanate sulforaphane (SFN) is an acknowledged cancer chemopreventive agent with multiple blocking and suppressive activities. It has been reported that SFN might act by inhibiting histone deacetylation. Since we showed before that deacetylase inhibitors such as trichostatin A (TSA) can inhibit STAT5-mediated transcription [21], we investigated here whether SFN can also inhibit STAT5 activity, possibly via inhibition of deacetylase activity. We now show that, similarly to TSA, SFN treatment reduces the expression of STAT5 target genes at the RNA level in normal and cancer cells. Like TSA, SFN does not target STAT5 phosphorylation or binding of activated STAT5 to DNA, supporting a model in which both small-molecule inhibitors target STAT5 transcriptional activity (Figure 8). Unlike TSA however, SFN only modestly affected the recruitment of RNA polymerase II to the promoter of STAT5 target genes. Importantly, as opposed to TSA, no significant changes in histone acetylation were noted in cells treated with SFN, neither globally nor locally at specific promoters. Our data therefore suggest that inhibition of STAT5-mediated transcription by SFN is independent of its activity as deacetylase inhibitor.

Our finding that histone acetylation was not changed upon SFN treatment was unexpected and might appear inconsistent with published reports of SFN-induced increase in histone acetylation both globally and at specific promoters such as *p21* [17], [18], [20]. We believe that this apparent discrepancy might be due partly to the difference in treatment duration and to the cellular context in both types of studies. Given that our study is focusing on the short-term effect of SFN on the regulation of STAT5 activity, exposure of cells to SFN was limited to 60-90 minutes for gene expression analyses and 4 hours for global histone acetylation determination in Ba/F3 cells. The investigation of histone acetylation levels by Myzak and colleagues was performed following 47 hours of treatment with 15 µM SFN [17], [18]. Longer treatment might be necessary for sufficient accumulation of the SFN metabolites SFN-cysteine and SFN-*N*-acetylcysteine, the active histone deacetylase inhibitors, via the mercapturic acid pathway [15], [17]. This would explain why we observed no immediate effect of SFN on histone acetylation levels, as opposed to that of TSA. In support of this hypothesis, a 48-hour treatment of Ba/F3 cells with 10 µM SFN led to a 3.4-fold increase in histone H3 acetylation, although histone H4 acetylation was not increased in the same conditions.

On the other hand, a 48-hour treatment of Ba/F3 cells with 10 nM TSA resulted in a 44- and 25-fold increase in histone H3 and H4 acetylation respectively. This major difference in the effect of TSA and SFN in Ba/F3 cells contrasts with the observations made in the human prostate cell lines BPH-1, LnCaP and PC-3 and in the HEK 293 cells, showing comparable effects of TSA and SFN on histone acetylation, in that case using TSA concentrations 30-times higher than in the present study [17], [18]. This strongly suggests that part of the activity of SFN as a deacetylase inhibitor is cell type-dependent and might not be highly relevant in Ba/F3 cells. We cannot exclude at this point that SFN alters STAT5 activity by modulating acetylation of non-histone proteins. Notably, STAT5 proteins can be acetylated on specific lysine residues, hence modifying their transcriptional activity [28], [29]. Recent data from our laboratory suggest, however, that acetylation of STAT5 does not modulate its activity in Ba/F3 cells (manuscript in preparation), making it unlikely that SFN-mediated inhibition of STAT5 activity involves direct alteration of its acetylation status.

Nonetheless, our data revealed a novel function of SFN as STAT5 inhibitor, possibly targeting its transcriptional activity at a step following binding of activated STAT5 to DNA. The question remains as to how SFN exerts this immediate inhibitory activity. Sulforaphane is an electrophile that can potentially react with thiols. SFN was shown to react with free sulfhydryl groups of cysteine residues in a number of proteins and, in some cases, to directly alter their function [8], [10], [11], [83]. Therefore, it remains possible that SFN reacts with cysteine residues within STAT5 or a cofactor of STAT5, thereby affecting STAT5-mediated transcription. On the other hand, it was shown that the uptake and accumulation of SFN in the cell occur through conjugation with intracellular glutathione (GSH), resulting in a transient drop in intracellular GSH [12]. Other electrophilic natural compounds, such as terpenes and chalcones, are known to react with and provoke a temporary decrease in intracellular GSH. The resulting mild oxidative stress was shown to trigger S-glutathionylation of cysteines within transcription factors such as STAT3 or NF-κB, thereby inhibiting their activity [84]–[87]. It is therefore tempting to speculate that SFN, as an electrophile, might as well inhibit the activity of STAT5 or of a STAT5-associated factor important for Pol II recruitment and/or promoter clearance [82] via S-glutathionylation. Further investigations will be necessary to address these potential thiol-dependent activities.

It should be noted that, since the consequences of SFN treatment on RNA polymerase II occupancy at the STAT5 target genes investigated were not dramatic, we cannot yet exclude the possibility that the observed decrease in STAT5 target gene mRNA levels is the result of a post-transcriptional - rather than transcriptional - effect. In support of this hypothesis, gene expression profiling of SFN-treated human prostate cancer cells identified an enrichment in genes involved in RNA post-transcriptional modification [88].

We showed that expression of *JunB*, was also inhibited by SFN in Ba/F3 cells stimulated with IL-3. By contrast, the deacetylase inhibitor TSA did not affect expression of *JunB*, as previously reported [21]. *JunB* and *c-Fos* are known MAPK-regulated genes [75], [76]. Accordingly, expression of *JunB* and *c-Fos* was induced in IL-3-stimulated Ba/F3 cells but not in Ba/F3-1*6 cells in the absence of JAK2/MAPK activation. A variety of opposite effects of SFN on the regulation of the MAPK pathway has been reported, mostly depending on the cell lines used and the concentration of SFN applied [5], [89]–[94]. However, since *c-Fos* expression was not affected by SFN in IL-3-stimulated Ba/F3 cells, it is unlikely that SFN inhibits the JAK2/MAPK pathway in Ba/F3 cells. Moreover, basal expression of *JunB* in Ba/F3-1*6 cells was also down-regulated by SFN, suggesting that the activity of another factor involved in *JunB* basal transcription is targeted by SFN. Beside the MAPK pathway, *JunB* expression is regulated by various signaling pathways and transcription factors [95]–[99]. In line with the proposed activity of SFN as an electrophile, it is envisageable that SFN alters the activity of a factor essential for *JunB* - but not *c-Fos* - expression.

In conclusion, we identified SFN as a novel STAT5 inhibitor, likely targeting STAT5-mediated transcription independently of its proposed action as an inhibitor of histone deacetylation (Figure 8). Interestingly, the inhibitory effect of SFN was also demonstrated in cell lines transformed by constitutive active STAT5, therefore suggesting a beneficial role of the natural isothiocyanate SFN not only in cancer prevention but also for patients with STAT5-associated cancers.

## Supporting Information

Figure S1 Effect of SFN treatment on Ba/F3, Ba/F3-1*6 and K562 cell death. Growing Ba/F3, Ba/F3-1*6 and K562 cells were incubated for 24 and 48 hours in the presence of the indicated concentrations of TSA and SFN. Cell death was measured by Trypan Blue exclusion assay and was expressed as the percentage of dead cells. The number of living cells at 24 and 48 hours of treatment from the same experiment is presented in Figure 2B.
(TIF)

Figure S2 **Schematic representation of the genes and PCR amplicons investigated by chromatin immunoprecipitation.** The STAT5 target genes *Cis* and *Osm* carry four and two STAT5 binding sites within their proximal promoters respectively. Amplicons A (−188/−104) and I (−184/−122) overlapping the STAT5 binding sites of *Cis* and *Osm* respectively served for the detection of the chromatin co-precipitated with STAT5 antibodies. Amplicons B (−18/+55) and J (+25/+87) overlapping the transcription start sites of *Cis* and *Osm* respectively served for the detection of the chromatin co-precipitated with RNA polymerase II antibodies. Amplicons B (*Cis*), J (*Osm*) and K (*p21*; −120/−61) were used following chromatin immunoprecipitation with histone-specific (Ac-H3, Ac-H4, H3) antibodies. Additional *Cis* amplicons are shown in Figure 5D. The transcribed regions (dark grey arrow) of *Cis*, *Osm* and *p21* are not represented at their respective proportional scale.
(TIF)

Figure S3 **In contrast to TSA, SFN does not prevent recruitment of RNA polymerase II to the promoter of STAT5 target genes.** Ba/F3 cells were pre-treated 30 minutes with DMSO (vehicle), 0.2 µM TSA or 10 µM SFN and further stimulated 30 minutes with 5 ng/mL IL-3. Chromatin immunoprecipitation (ChIP) was performed as described in Materials and Methods using antibodies directed against STAT5 or RNA polymerase II (RNA Pol II) proteins. Co-precipitated genomic DNA was analyzed by quantitative PCR using primers specific for the STAT5 binding sites (STAT5 ChIP; amplicons A and I in Figure S2) or the transcription start site (RNA Pol II ChIP; amplicons B and J in Figure S2) of the mouse *Cis* (**A**) and *Osm* (**B**) genes. While TSA treatment prevents recruitment of RNA polymerase II following STAT5 binding to DNA, in agreement with our published data [21], SFN treatment has only partial (*Osm*) or no (*Cis*) effect on RNA polymerase II occupancy at the transcription start site of STAT5 target genes. Two-tailed paired Student's t-test, SFN-treated and TSA-treated compared to vehicle control (IL-3-stimulated); *P<0.05, **P<0.005, ***P<0.001, ****P<0.0001; ns, not significant.
(TIF)

Figure S4 **RNA polymerase II occupancy along the** ***Cis*** **open reading frame is reproducibly reduced in SFN-treated cells.** Ba/F3 cells were pre-treated 30 minutes with DMSO (vehicle), 10 µM (**A**) or 20 µM (**B**) SFN and further stimulated with 5 ng/mL IL-3 for 30 minutes. Chromatin immunoprecipitation (ChIP) was performed as described above using antibodies directed against RNA polymerase II (RNA Pol II). Co-precipitated genomic DNA was analyzed by quantitative PCR using primers spanning the open reading frame of the *Cis* gene (amplicons C-H, as schematized in the upper panel). Panels **A** and **B** represent data from two independent experiments. Data from panel **B** are the same as shown in figure 5B. Two-tailed paired Student's t-test, SFN-treated compared to vehicle control (IL-3-stimulated); *P* values and their significance are indicated above each pair; ns, not significant.
(TIF)

Figure S5 **Prolonged treatment of Ba/F3 cells with SFN results in increased histone H3 acetylation.** Ba/F3 cells were treated for the indicated times with either 10 nM TSA or 10 µM SFN. Whole-cell Freeze-Thaw protein lysates were analyzed by Western blot using antibodies specific for acetylated histone H3 (Ac-H3) and H4 (Ac-H4) and for total histone H3 proteins, as in Figure 6. To allow an accurate assessment of histone acetylation levels, Western blots were repeated 4 times and chemiluminescence signals were quantified using ImageQuant TL (GE Healthcare). Ac-H3 and Ac-H4 signals were normalized to total H3 and expressed relative to the untreated control (arbitrarily set to 1; see values below each lane) (**A**). Means ±SD of relative Ac-H3/H3 and Ac-H4/H3 values (fold of untreated control) from the 4 blots shown in (A) are depicted in (**B**). Two-tailed paired Student's t-test, SFN-treated compared to untreated control; *P<0.05. Treatment of Ba/F3 cells up to 48 hours with SFN resulted in a global increase in acetylated histone H3 (3.4-fold) while acetylated histone H4 level was slightly decreased (1.6-fold).
(TIF)

Figure S6 **SFN treatment does not affect histone acetylation at the promoters of STAT5 target (** ***Cis, Osm*** **) and control (** ***p21*** **) genes (% input DNA).** Ba/F3 cells were pre-treated 30 minutes with DMSO (vehicle), 0.2 µM TSA or 10 µM SFN and further stimulated 30 minutes with 5 ng/mL IL-3. Chromatin immunoprecipitation (ChIP) was performed using antibodies directed against acetylated histone H3 (Ac-H3) and H4 (Ac-H4) and against histone H3 proteins (total H3). Co-precipitated genomic DNA was analyzed by quantitative PCR using primers specific for the transcription start sites of the mouse *Cis* (**A**) and *Osm* (**B**) genes (amplicons B and J respectively in Figure S2), as well as for the proximal promoter region of the mouse *p21* gene (amplicon K in Figure S2) as a control (**C**). Ac-H3 and Ac-H4 ChIP data normalized to total Histone H3 are shown in Figure 7.
(TIF)

File S1 **Raw data (Quantitative PCR CT values, WST-1 OD values).**
(PDF)
