# Supplementary figures and images for: The Natural Chemopreventive Agent Sulforaphane Inhibits STAT5 Activity
Source: PLoS One. 2014 Jun 9;9(6):e99391. doi: 10.1371/journal.pone.0099391 (PMC4051870; doi:10.1371/journal.pone.0099391)

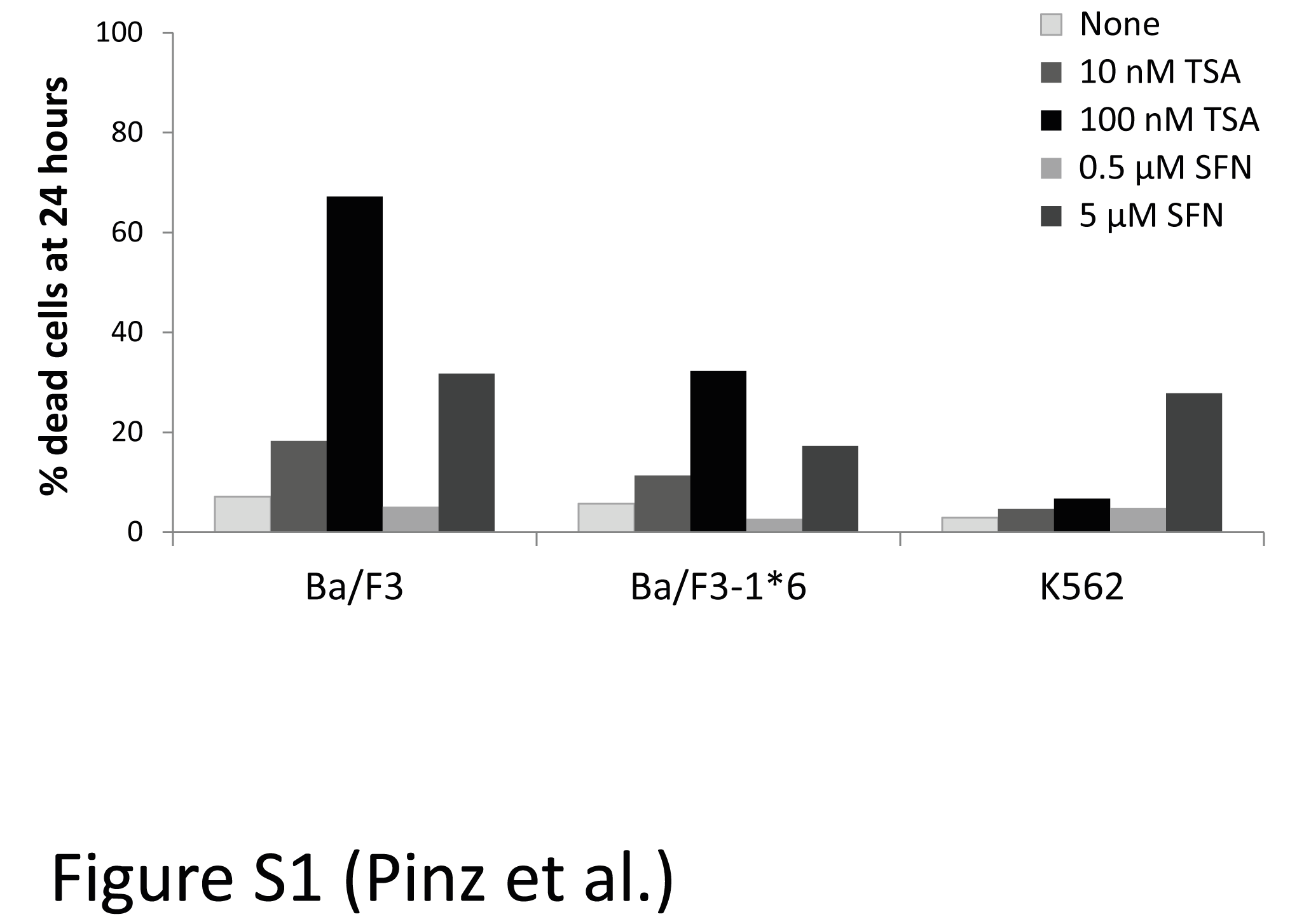

Supplement: Figure S1 — Effect of SFN treatment on Ba/F3, Ba/F3-1*6 and K562 cell death. Growing Ba/F3, Ba/F3-1*6 and K562 cells were incubated for 24 and 48 hours in the presence of the indicated concentrations of TSA and SFN. Cell death was measured by Trypan Blue exclusion assay and was expressed as the percentage of dead cells. The number of living cells at 24 and 48 hours of treatment from the same experiment is presented in Figure 2B. (TIF) [file pone.0099391.s001.tif]

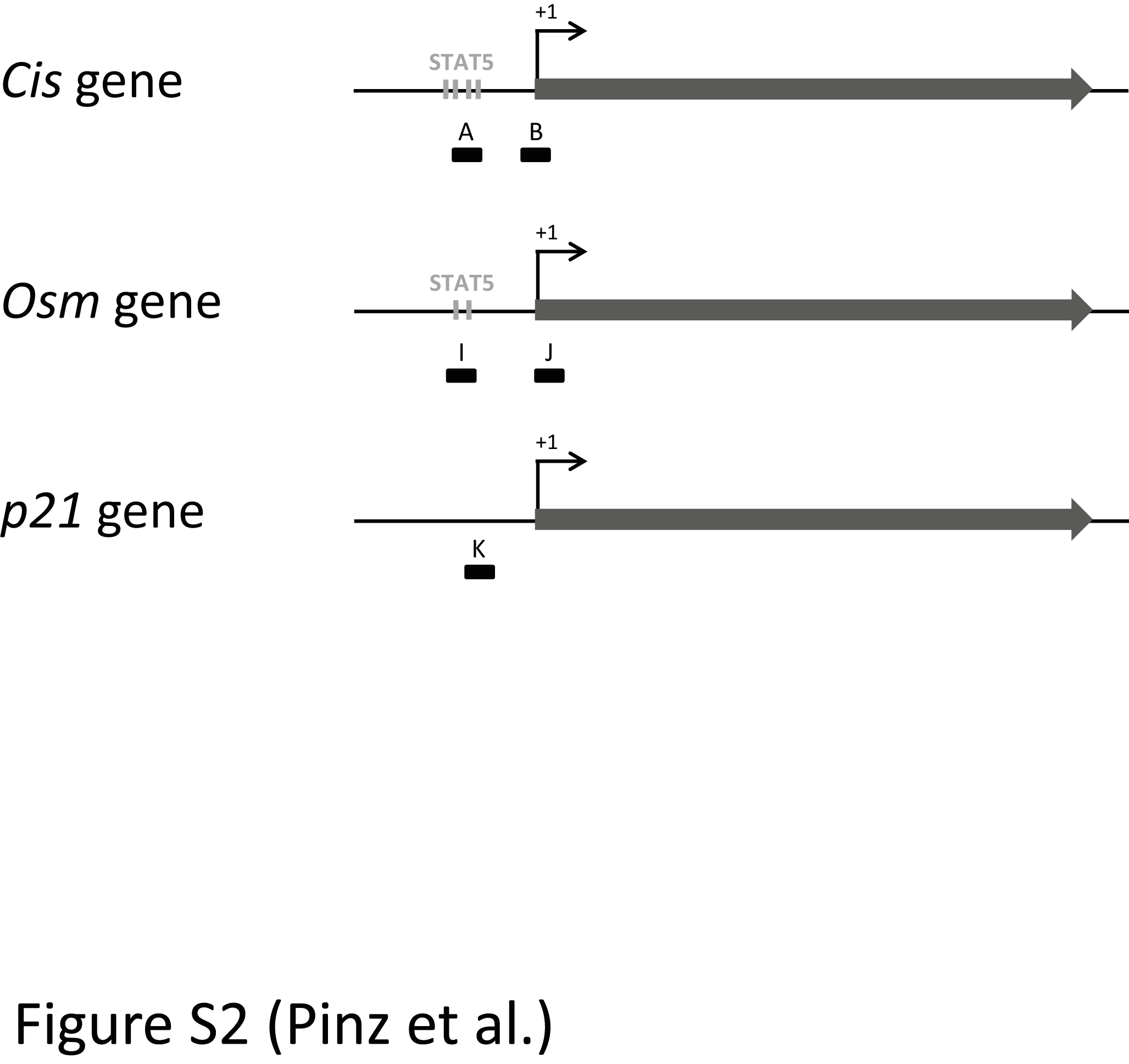

Supplement: Figure S2 — Schematic representation of the genes and PCR amplicons investigated by chromatin immunoprecipitation. The STAT5 target genes Cis and Osm carry four and two STAT5 binding sites within their proximal promoters respectively. Amplicons A (−188/−104) and I (−184/−122) overlapping the STAT5 binding sites of Cis and Osm respectively served for the detection of the chromatin co-precipitated with STAT5 antibodies. Amplicons B (−18/+55) and J (+25/+87) overlapping the transcription start sites of Cis and Osm respectively served for the detection of the chromatin co-precipitated with RNA polymerase II antibodies. Amplicons B (Cis), J (Osm) and K (p21; −120/−61) were used following chromatin immunoprecipitation with histone-specific (Ac-H3, Ac-H4, H3) antibodies. Additional Cis amplicons are shown in Figure 5D. The transcribed regions (dark grey arrow) of Cis, Osm and p21 are not represented at their respective proportional scale. (TIF) [file pone.0099391.s002.tif]

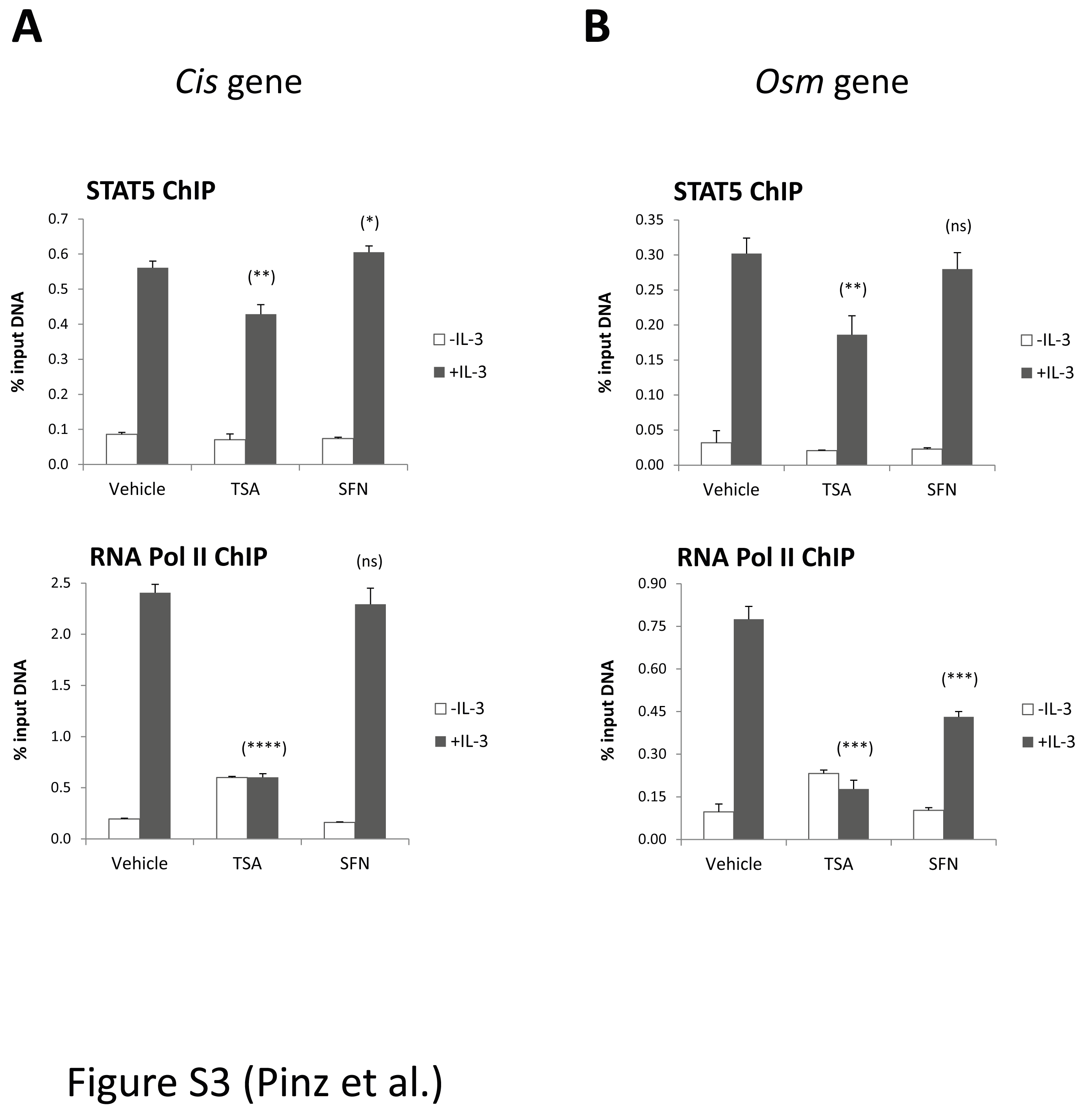

Supplement: Figure S3 — In contrast to TSA, SFN does not prevent recruitment of RNA polymerase II to the promoter of STAT5 target genes. Ba/F3 cells were pre-treated 30 minutes with DMSO (vehicle), 0.2 µM TSA or 10 µM SFN and further stimulated 30 minutes with 5 ng/mL IL-3. Chromatin immunoprecipitation (ChIP) was performed as described in Materials and Methods using antibodies directed against STAT5 or RNA polymerase II (RNA Pol II) proteins. Co-precipitated genomic DNA was analyzed by quantitative PCR using primers specific for the STAT5 binding sites (STAT5 ChIP; amplicons A and I in Figure S2) or the transcription start site (RNA Pol II ChIP; amplicons B and J in Figure S2) of the mouse Cis (A) and Osm (B) genes. While TSA treatment prevents recruitment of RNA polymerase II following STAT5 binding to DNA, in agreement with our published data [21], SFN treatment has only partial (Osm) or no (Cis) effect on RNA polymerase II occupancy at the transcription start site of STAT5 target genes. Two-tailed paired Student's t-test, SFN-treated and TSA-treated compared to vehicle control (IL-3-stimulated); *P<0.05, **P<0.005, ***P<0.001, ****P<0.0001; ns, not significant. (TIF) [file pone.0099391.s003.tif]

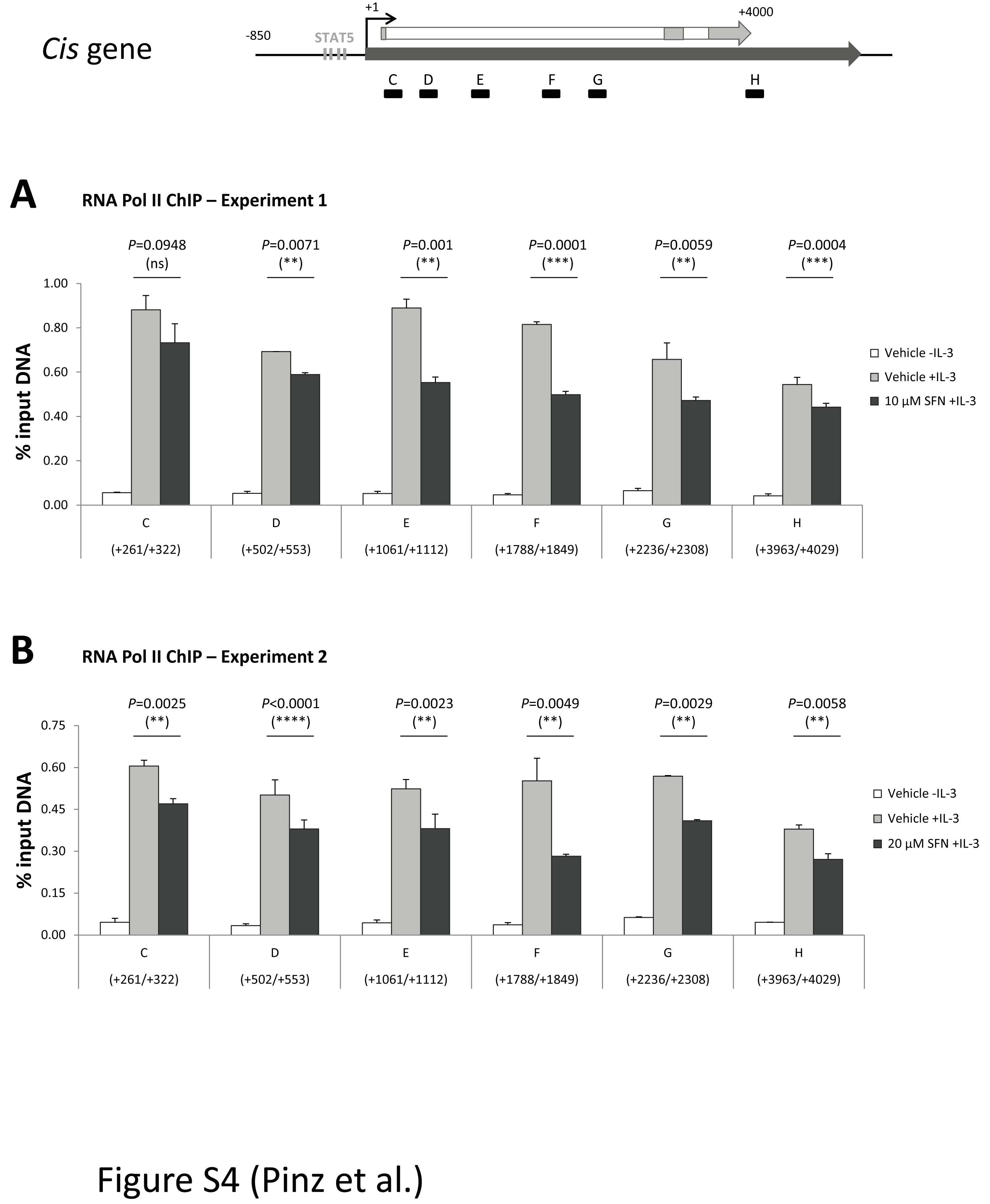

Supplement: Figure S4 — RNA polymerase II occupancy along the Cis open reading frame is reproducibly reduced in SFN-treated cells. Ba/F3 cells were pre-treated 30 minutes with DMSO (vehicle), 10 µM (A) or 20 µM (B) SFN and further stimulated with 5 ng/mL IL-3 for 30 minutes. Chromatin immunoprecipitation (ChIP) was performed as described above using antibodies directed against RNA polymerase II (RNA Pol II). Co-precipitated genomic DNA was analyzed by quantitative PCR using primers spanning the open reading frame of the Cis gene (amplicons C-H, as schematized in the upper panel). Panels A and B represent data from two independent experiments. Data from panel B are the same as shown in figure 5B. Two-tailed paired Student's t-test, SFN-treated compared to vehicle control (IL-3-stimulated); P values and their significance are indicated above each pair; ns, not significant. (TIF) [file pone.0099391.s004.tif]

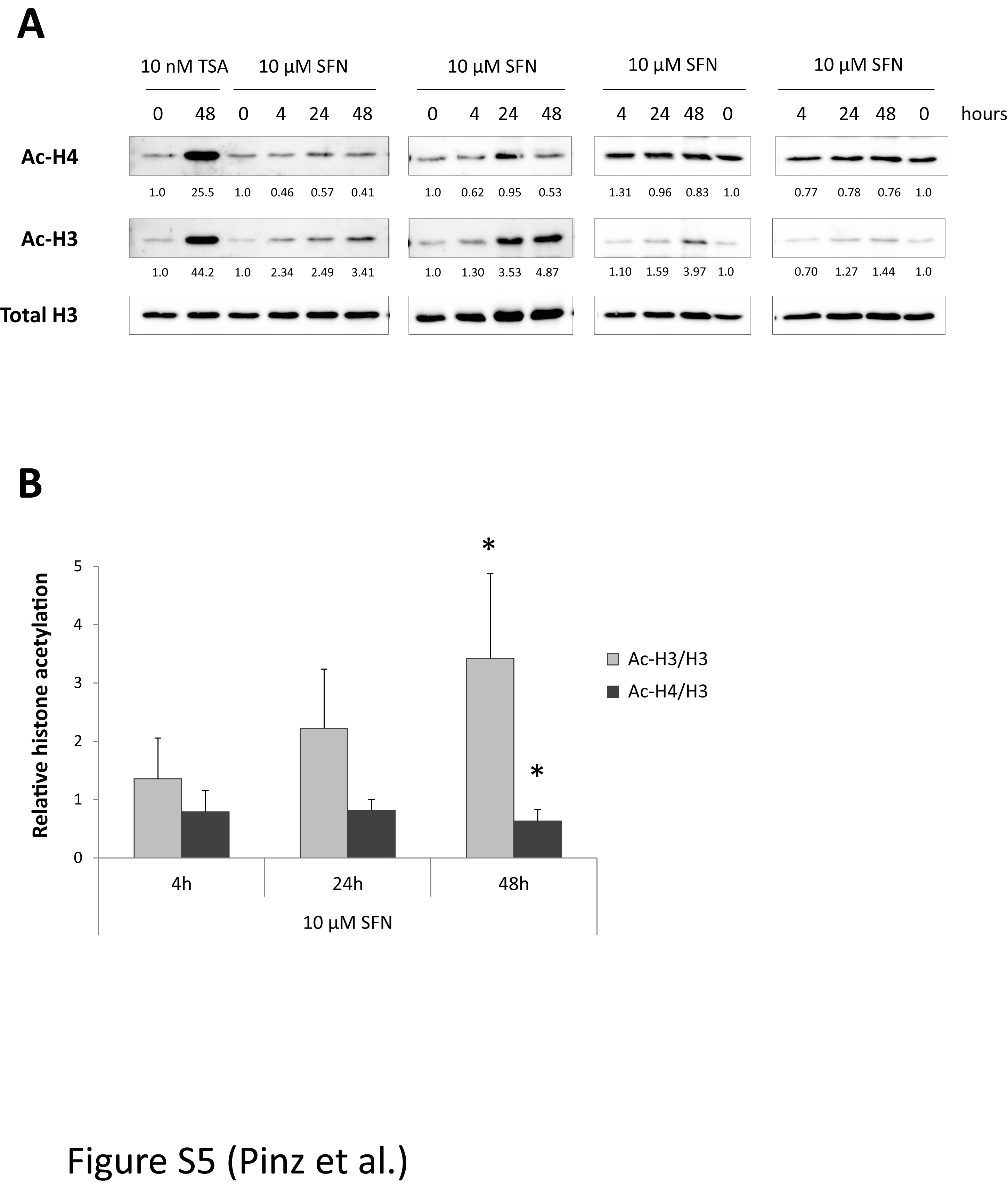

Supplement: Figure S5 — Prolonged treatment of Ba/F3 cells with SFN results in increased histone H3 acetylation. Ba/F3 cells were treated for the indicated times with either 10 nM TSA or 10 µM SFN. Whole-cell Freeze-Thaw protein lysates were analyzed by Western blot using antibodies specific for acetylated histone H3 (Ac-H3) and H4 (Ac-H4) and for total histone H3 proteins, as in Figure 6. To allow an accurate assessment of histone acetylation levels, Western blots were repeated 4 times and chemiluminescence signals were quantified using ImageQuant TL (GE Healthcare). Ac-H3 and Ac-H4 signals were normalized to total H3 and expressed relative to the untreated control (arbitrarily set to 1; see values below each lane) (A). Means ±SD of relative Ac-H3/H3 and Ac-H4/H3 values (fold of untreated control) from the 4 blots shown in (A) are depicted in (B). Two-tailed paired Student's t-test, SFN-treated compared to untreated control; *P<0.05. Treatment of Ba/F3 cells up to 48 hours with SFN resulted in a global increase in acetylated histone H3 (3.4-fold) while acetylated histone H4 level was slightly decreased (1.6-fold). (TIF) [file pone.0099391.s005.tif]

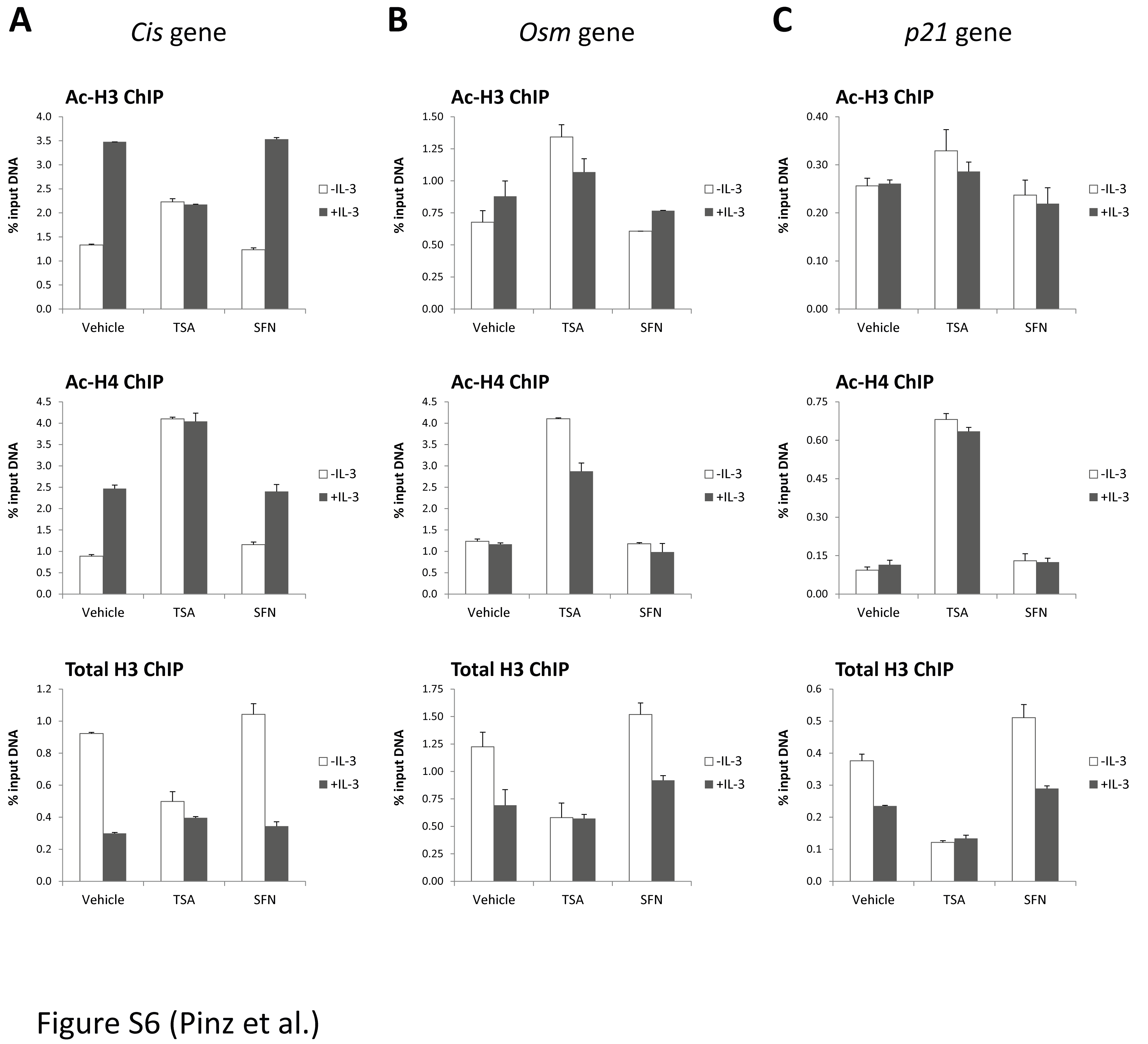

Supplement: Figure S6 — SFN treatment does not affect histone acetylation at the promoters of STAT5 target ( Cis, Osm ) and control ( p21 ) genes (% input DNA). Ba/F3 cells were pre-treated 30 minutes with DMSO (vehicle), 0.2 µM TSA or 10 µM SFN and further stimulated 30 minutes with 5 ng/mL IL-3. Chromatin immunoprecipitation (ChIP) was performed using antibodies directed against acetylated histone H3 (Ac-H3) and H4 (Ac-H4) and against histone H3 proteins (total H3). Co-precipitated genomic DNA was analyzed by quantitative PCR using primers specific for the transcription start sites of the mouse Cis (A) and Osm (B) genes (amplicons B and J respectively in Figure S2), as well as for the proximal promoter region of the mouse p21 gene (amplicon K in Figure S2) as a control (C). Ac-H3 and Ac-H4 ChIP data normalized to total Histone H3 are shown in Figure 7. (TIF) [file pone.0099391.s006.tif]
