# Supplementary material for: The Natural Chemopreventive Agent Sulforaphane Inhibits STAT5 Activity
Source: PLoS One. 2014 Jun 9;9(6):e99391. doi: 10.1371/journal.pone.0099391 (PMC4051870; doi:10.1371/journal.pone.0099391)
Supplement: File S1 — Raw data (Quantitative PCR CT values, WST-1 OD values). (PDF) [file pone.0099391.s007.pdf]

Pinz et al. - Supporting File S1 (Raw data)

Figure 1B - CT values

	Ba/F3					
	#1	#2	#3	#4	#5	#6
	**- IL-3**	**+ IL-3**				
	**DMSO**	**DMSO**	**TSA**	**SFN**		
	**0.02%**	**0.02%**	**200nM**	**0.4µM**	**2µM**	**10µM**
**mCis**	25.94	22.42	23.44	22.37	22.81	23.55
**mCis**	26.75	22.48	24.01	22.36	22.89	23.21
**mCis**	26.99	22.23	23.66	22.33	22.72	23.57
**c-Myc**	22.63	21.39	24.14	21.48	21.59	22.64
**c-Myc**	22.53	21.63	24.42	21.5	21.72	22.41
**c-Myc**	22.63	21.19	23.94	21.3	21.81	22.64
**Osm**	28.63	21.6	25.57	21.77	22.5	24.05
**Osm**	27.45	21.56	25.55	21.82	22.36	24.26
**Osm**	28.46	21.58	24.75	21.53	22.16	24.55
**36b4**	17.24	17.21	17.21	17.23	17.2	17.35
**36b4**	17.08	17.2	17.18	17.19	17.34	17.26
**S9**	18.47	18.61	18.34	18.18	18.43	18.43
**S9**	18.38	18.56	18.4	18.58	18.58	18.64
**S9**	18.47	18.42	18.34	18.48	18.25	18.41
**S9**	18.5	18.58	18.66	18.73	18.62	18.51
**S9**	18.43	18.51	18.47	18.5	18.7	18.61

Figure 3 - CT values

	Ba/F3				Ba/F3-1*6 F7		
	#1	#2	#3	#4	#1	#2	#3
	**- IL-3**	**+ IL-3**					
	**DMSO**	**DMSO**	**TSA**	**SFN**	**DMSO**	**TSA**	**SFN**
	**0.02%**	**0.02%**	**200nM**	**10µM**	**0.02%**	**200nM**	**10µM**
**mCis**	26.58	21.25	23.31	21.88	21.97	23.54	23.08
**mCis**	26.59	20.97	23.44	22.06	22.05	23.61	23.18
**c-Myc**	25.09	21.21	24.69	22.5	19.58	23.02	22.07
**c-Myc**	23.5	21.19	24.36	22.64	19.94	23.29	22.11
**Pim-1**	27.59	21.62	23.58	22.76	20.69	23.3	22.8
**Pim-1**	26.86	21.8	23.72	22.91	20.78	23.4	22.82
**Pim-1**	26.15	21.7	23.53	22.7	20.78	23.14	22.61
**SOCS-1**	31.32	24.75	26.06	25.91	23.8	26	27.38
**SOCS-1**	-	24.61	26.23	26.38	24.18	25.28	26.49
**Osm**	26.77	22.96	24.79	25.7	23.49	25.44	25.11
**Osm**	27	22.64	24.31	25.18	23.1	25.22	24.43
**JunB**	25.96	22.33	22.3	23.5	24.37	24.74	27.43
**JunB**	25.78	22.29	22.6	23.59	24.77	24.43	26.41
**c-Fos**	29.51	25.11	23.67	24.94	-	27.4	29.64
**c-Fos**	29.93	24.9	23.8	25.24	29.34	28.35	37.34
**c-Fos**	29.91	25.05	23.69	25.49			
**36b4**	17.24	17.11	17.14	17.26	16.01	16.43	16.14
**36b4**	17.29	17.02	17.07	17.3	15.82	16.37	16.21
**36b4**	17.36	17.15	17.14	17.3	15.77	16.41	16.06
**S9**	18.36	18.53	18.52	18.51	17.67	17.86	17.64
**S9**	18.38	18.42	18.28	18.56	17.53	17.76	17.56
**S9**	18.31	18.38	18.38	18.57	17.34	17.68	17.52
**S9**	18.45	18.37	18.35	18.51	17.54	17.69	17.49

	K562			
	#1	#2	#3	#4
	**DMSO**	**Imatinib**	**TSA**	**SFN**
	**0.02%**	**1uM**	**200nM**	**10uM**
**hCis**	24.57	26.4	24.45	24.76
**hCis**	24.28	26.21	24.26	24.46
**c-Myc**	20.64	22.29	21.09	21.56
**c-Myc**	20.56	22.54	20.9	22.04
**LMNA**	20.66	20.38	19.64	20.32
**LMNA**	20.44	20.35	19.56	20.2

Figure 5 - CT values

Figure 5A - mRNA levels

	#1	#2	#3	#4	#5	#6
	**0.02% DMSO**		**10µM SFN**		**20µM SFN**	
	**-IL-3**	**+IL-3**	**-IL-3**	**+IL-3**	**-IL-3**	**+IL-3**
**Cis**	26.22	21.94	27.12	22.54	28.05	23.46
**Cis**	26.5	22.04	26.53	22.36	26.25	23.41
**Osm**	27.12	21.56	27.5	23.16	26.84	25.3
**Osm**	26.84	21.73	28.75	23.17	27.53	25.14
**S9**	18.23	18.21	18.47	18.3	18.5	18.35
**S9**	18.3	18.16	18.47	18.14	18.58	18.45

Figure 5B-C - ChIP - *Cis* gene

	#1	#2	#3	#4	#5	#6
	**0.02% DMSO**		**10µM SFN**		**20µM SFN**	
Amplicon A	**-IL-3**	**+IL-3**	**-IL-3**	**+IL-3**	**-IL-3**	**+IL-3**
STAT5	24.21	22.12	24.52	21.83	22.72	24.54
STAT5	24.76	22.16	24.26	22.09	22.35	25.17
STAT5	24.46	22.2	23.78	22	22.48	24.68
Input	19.97	20.1	19.9	20.01	20.24	20
Input	19.83	20.11	19.9	19.84	20.15	*22.62
Input	19.79	20.11	19.91	19.92	20.54	20.09

* outlier (excluded from average calculation)

Amplicon B						
RNA Pol II	23.43	20.42	23.8	20	20.32	23.66
RNA Pol II	23.31	20.41	23.9	20.03	20.23	23.73
RNA Pol II	23.44	20.46	23.66	20.07	20.45	23.65
Input	20.16	20.56	20.21	20.44	20.46	20.11
Input	20.27	20.74	20.32	20.46	21.04	20.15
Input	20.19	20.52	20.05	20.48	20.34	20.12

Figure 5B-C - ChIP - *Osm* gene

	#1	#2	#3	#4	#5	#6
	**0.02% DMSO**		**10µM SFN**		**20µM SFN**	
Amplicon I	**-IL-3**	**+IL-3**	**-IL-3**	**+IL-3**	**-IL-3**	**+IL-3**
STAT5	26.31	23.53	26.16	23.33	26.33	24.25
STAT5	26.86	23.55	26.71	23.56	27.33	24.34
Input	20.24	20.36	20.1	20.02	20.17	20.54
Input	20.38	20.2	20.18	20.09	20.56	20.3
Amplicon J						
RNA Pol II	25.05	23.44	25.36	23.47	26.61	24.57
RNA Pol II	25.31	23.67	25.45	23.48	26.35	24.41
Input	21.16	21.08	21.07	21.24	21.06	21.33
Input	21.06	21.08	21.07	21.16	21.06	21.13

Figure 5D [and S4 Experiment 2 (B)] - CT values - RNA Pol II ChIP - *Cis* gene

	Amplicon B			
	#1	#2	#3	
	0.02% DMSO		20µM SFN	
	-IL-3	+IL-3	+IL-3	
RNA Pol II	23.8	20.75	20.68	
RNA Pol II	23.52	20.62	*20.86	* outlier (excluded from calculation)
RNA Pol II	23.43	20.42	20.32	
RNA Pol II	23.31	20.41	20.23	
RNA Pol II	23.44	20.46	20.45	
Input	20.13	20.47	20.53	
Input	20.14	20.47	20.42	
Input	20.18	20.53	20.4	
Input	20.14	20.51	20.39	
Input	20.16	20.56	20.46	
Input	20.27	20.74	*21.04	* outlier (excluded from average calculation)
Input	20.19	20.52	20.34	

	Amplicon C			Amplicon D		
	#1	#2	#3	#1	#2	#3
	0.02% DMSO		20µM SFN	0.02% DMSO		20µM SFN
	-IL-3	+IL-3	+IL-3	-IL-3	+IL-3	+IL-3
RNA Pol II	25.11	22.04	22.31	26.16	22.45	22.87
RNA Pol II	25.94	22.11	22.38	26.03	22.67	22.94
Input	20.1	20.26	20.01	20.39	20.4	20.43
Input	20.17	20.33	20.02	20.26	20.44	20.28
Input	19.97	20.13	20.24	20.21	20.37	20.34
Input	20	20.08	20.21	20.27	20.43	20.37

	Amplicon E			Amplicon F		
	#1	#2	#3	#1	#2	#3
	0.02% DMSO		20µM SFN	0.02% DMSO		20µM SFN
	-IL-3	+IL-3	+IL-3	-IL-3	+IL-3	+IL-3
RNA Pol II	25.52	21.78	22.25	25.84	21.82	23.05
RNA Pol II	25.06	21.91	22.53	25.43	22.12	23
Input	19.76	19.82	19.84	19.95	19.98	20.07
Input	19.47	19.7	19.84	19.47	19.93	20.03

	Amplicon G			Amplicon H		
	#1	#2	#3	#1	#2	#3
	0.02% DMSO		20µM SFN	0.02% DMSO		20µM SFN
	-IL-3	+IL-3	+IL-3	-IL-3	+IL-3	+IL-3
RNA Pol II	25.8	22.66	23.14	25.83	22.84	23.24
RNA Pol II	25.88	22.67	23.16	25.81	22.76	23.39
Input	21.04	20.68	20.79	20.42	20.28	20.27
Input	20.37	20.72	20.63	20.03	20.22	20.29

Figure S3 - CT values

ChIP - *Cis* gene

	#1	#2	#3	#4	#5	#6
	**0.02% DMSO**		**200nM TSA**		**10µM SFN**	
Amplicon A	**-IL-3**	**+IL-3**	**-IL-3**	**+IL-3**	**-IL-3**	**+IL-3**
STAT5	25.38	22.77	25.4	23.09	25.82	22.77
STAT5	25.51	22.7	25.87	23.22	25.7	22.71
Input	20.72	20.77	20.57	20.74	20.73	20.84
Input	20.78	20.73	20.72	20.82	20.96	20.89
Amplicon B						
RNA Pol II	24.37	21.13	22.71	22.73	24.71	21.18
RNA Pol II	24.46	21.06	22.67	22.85	24.64	21.04
Input	20.85	21.18	20.86	20.94	20.97	21.1
Input	20.95	21.24	20.74	20.87	20.8	21.21

ChIP - *Osm* gene

	#1	#2	#3	#4	#5	#6
	**0.02% DMSO**		**200nM TSA**		**10µM SFN**	
Amplicon I	**-IL-3**	**+IL-3**	**-IL-3**	**+IL-3**	**-IL-3**	**+IL-3**
STAT5	25.76	22.89	26.78	23.89	26.87	23.18
STAT5	26.93	23.04	26.87	23.59	26.71	23.01
Input	20.1	20.1	20.09	20.17	20.17	20.04
Input	20.11	20.07	20.06	20.14	20.22	20.17
Amplicon J	25.9	22.62	24.25	24.95	25.68	23.45
RNA Pol II	25.3	22.74	24.36	24.59	25.86	23.54
RNA Pol II	21.06	21.12	21.09	21.14	21.24	21.11
Input	21.04	21.2	21	21.09	21.43	21.15
Input	21.05	21.16	21.05	21.12	21.34	21.13

Figure S4 - CT values - RNA Pol II ChIP - *Cis* gene - Experiment 1 (A)

	Amplicon C		
	#1	#2	#3
	**0.02% DMSO**		**20µM SFN**
	**-IL-3**	**+IL-3**	**+IL-3**
RNA Pol II	25.43	21.4	21.96
RNA Pol II	25.36	21.55	21.72
Input	20.18	20.13	20.24
Input	20.01	20.15	20.23

Amplicon D		
#1	#2	#3
**0.02% DMSO**		**20µM SFN**
**-IL-3**	**+IL-3**	**+IL-3**
26.09	22.26	22.52
25.73	22.26	22.55
20.51	20.61	20.59
20.51	20.55	20.65

	Amplicon E		
	#1	#2	#3
	**0.02% DMSO**		**20µM SFN**
	**-IL-3**	**+IL-3**	**+IL-3**
RNA Pol II	25.27	21.46	22.1
RNA Pol II	25.64	21.55	22.01
Input	20.07	20.22	20.1
Input	20.01	20.15	20

Amplicon F		
#1	#2	#3
**0.02% DMSO**		**20µM SFN**
**-IL-3**	**+IL-3**	**+IL-3**
25.74	21.92	22.44
26.02	21.89	22.5
20.29	20.45	20.33
20.27	20.47	20.3

	Amplicon G		
	#1	#2	#3
	**0.02% DMSO**		**20µM SFN**
	**-IL-3**	**+IL-3**	**+IL-3**
RNA Pol II	25.93	22.54	22.9
RNA Pol II	25.6	22.31	22.83
Input	20.72	20.71	20.63
Input	20.61	20.62	20.63

Amplicon H		
#1	#2	#3
**0.02% DMSO**		**20µM SFN**
**-IL-3**	**+IL-3**	**+IL-3**
26.14	22.6	22.93
26.56	22.72	23.01
20.65	20.68	20.7
20.57	20.58	20.58

Figures 7 and S6 - CT values

ChIP - *Cis* gene

	#1	#2	#3	#4	#5	#6
	**0.02% DMSO**		**200nM TSA**		**10µM SFN**	
Amplicon B	**-IL-3**	**+IL-3**	**-IL-3**	**+IL-3**	**-IL-3**	**+IL-3**
Ac-H3	21.04	19.99	20.26	20.34	21.11	19.87
Ac-H3	21.01	19.99	20.2	20.35	21.17	19.89
Ac-H4	21.65	20.45	19.36	19.5	21.18	20.37
Ac-H4	21.57	20.52	19.34	19.4	21.29	20.51
H3	21.56	23.55	22.52	22.82	21.45	23.16
H3	21.55	23.51	22.27	22.78	21.32	23.33
Input	20.32	20.86	20.4	20.29	20.46	20.51
Input	20.31	20.55	20.25	20.44	20.25	20.68
Input	20.2	20.53	20.23	20.29	20.34	20.45
Input	20.32	20.61	20.06	20.23	20.12	20.56

ChIP - *Osm* gene

	#1	#2	#3	#4	#5	#6
	**0.02% DMSO**		**200nM TSA**		**10µM SFN**	
Amplicon J	**-IL-3**	**+IL-3**	**-IL-3**	**+IL-3**	**-IL-3**	**+IL-3**
Ac-H3	22.87	22.7	21.97	22.41	23.12	22.73
Ac-H3	23.14	22.42	21.81	22.21	23.12	22.72
Ac-H4	22.18	22.18	20.3	20.95	22.14	22.17
Ac-H4	22.09	22.12	20.31	20.81	22.19	22.59
H3	22.16	22.69	23.06	23.2	21.69	22.48
H3	21.99	22.79	22.87	23.12	21.88	22.39
H3	22.3	23.29	23.53	23.31	21.83	22.52
Input	21.21	21.03	21.24	21.22	21.36	21.08
Input	21.28	21.11	21.14	21.13	21.16	21.27
Input	21.37	21.51	21.28	21.4	21.18	21.22

ChIP - *p21* gene

	#1	#2	#3	#4	#5	#6
	**0.02% DMSO**		**200nM TSA**		**10µM SFN**	
Amplicon K	**-IL-3**	**+IL-3**	**-IL-3**	**+IL-3**	**-IL-3**	**+IL-3**
Ac-H3	23.88	*25.08	23.74	23.62	23.97	24.34
Ac-H3	24.05	23.77	23.44	23.8	24.28	24.18
Ac-H3	24	23.83	23.36	23.78	24.3	23.91
Ac-H4	25.55	24.92	*21.89	22.55	24.8	24.81
Ac-H4	25.53	25.27	22.49	22.62	24.97	25.17
Ac-H4	25.23	24.82	22.42	22.57	25.42	24.89
H3	23.51	*24.63	24.88	24.85	23.15	23.71
H3	23.41	23.94	25.01	24.93	22.94	23.7
H3	23.35	23.96	24.94	24.71	23.12	23.78
Input	20.79	20.68	20.69	20.78	20.99	20.85
Input	20.9	20.6	20.8	20.76	20.96	20.73
Input	20.89	20.85	20.76	20.78	20.89	20.79

* outliers (excluded from calculation)

Figures 2 - WST-1 assay - OD 450/620nm

Ba/F3 (0.75x10^5 cells; 5.5 h resting + 30 min drug pre-treatment + 90 min WST-1+IL3)

			TSA				SFN			
	**Triton-X100**	**DMSO 0.1%**	**1nM**	**10nM**	**100nM**	**1000nM**	**0.1µM**	**1µM**	**10µM**	**100µM**
450-620	0.092	1.134	0.973	0.993	0.996	1.012	0.963	1.059	0.991	0.683
	0.095	1.147	0.973	1.058	1.157	1.11	0.982	1.103	1.013	0.708
		1.139								
**Average**	**0.094**	**1.140**	**0.973**	**1.026**	**1.077**	**1.061**	**0.973**	**1.081**	**1.002**	**0.696**
**StDev**	**0.002**	**0.005**	**0.000**	**0.033**	**0.081**	**0.049**	**0.010**	**0.022**	**0.011**	**0.013**

Ba/F3-1*6 (0.75x10^5 cells; 30 min drug pre-treatment + 90 min WST-1)

			TSA				SFN			
	**Triton-X100**	**DMSO 0.1%**	**1nM**	**10nM**	**100nM**	**1000nM**	**0.1µM**	**1µM**	**10µM**	**100µM**
450-620	0.106	0.632	0.686	0.63	0.625	0.635	0.655	0.673	0.688	0.574
	0.11	0.604	0.668	0.688	0.649	0.672	0.66	0.674	0.701	0.572
**Average**	**0.108**	**0.618**	**0.677**	**0.659**	**0.637**	**0.654**	**0.658**	**0.674**	**0.695**	**0.573**
**StDev**	**0.002**	**0.014**	**0.009**	**0.029**	**0.012**	**0.019**	**0.003**	**0.001**	**0.007**	**0.001**

K562 (0.75x10^5 cells; 30 min drug pre-treatment + 90 min WST-1)

			TSA				SFN			
	**Triton-X100**	**DMSO 0.1%**	**1nM**	**10nM**	**100nM**	**1000nM**	**0.1µM**	**1µM**	**10µM**	**100µM**
450-620	0.177	1.168	1.085	1.056	1.091	1.119	1.105	1.113	1.01	0.811
	0.182	1.185	1.083	1.061	1.135	1.141	1.14	1.114	1.07	0.815
**Average**	**0.180**	**1.177**	**1.084**	**1.059**	**1.113**	**1.130**	**1.123**	**1.114**	**1.040**	**0.813**
**StDev**	**0.003**	**0.009**	**0.001**	**0.002**	**0.022**	**0.011**	**0.018**	**0.001**	**0.030**	**0.002**
